# Variation in the life history strategy underlies functional diversity of tumors

**DOI:** 10.1093/nsr/nwaa124

**Published:** 2020-06-05

**Authors:** Tao Li, Jialin Liu, Jing Feng, Zhenzhen Liu, Sixue Liu, Minjie Zhang, Yuezheng Zhang, Yali Hou, Dafei Wu, Chunyan Li, Yongbin Chen, Hua Chen, Xuemei Lu

**Affiliations:** State Key Laboratory of Genetic Resources and Evolution, Kunming Institute of Zoology, Chinese Academy of Sciences, Kunming 650223, China; CAS Key Laboratory of Genomic and Precision Medicine, Beijing Institute of Genomics, Chinese Academy of Sciences, Beijing 100101, China; Center for Excellence in Animal Evolution and Genetics, Chinese Academy of Sciences, Kunming 650223, China; University of Chinese Academy of Sciences, Beijing 100049, China; CAS Key Laboratory of Genomic and Precision Medicine, Beijing Institute of Genomics, Chinese Academy of Sciences, Beijing 100101, China; University of Chinese Academy of Sciences, Beijing 100049, China; State Key Laboratory of Genetic Resources and Evolution, Kunming Institute of Zoology, Chinese Academy of Sciences, Kunming 650223, China; CAS Key Laboratory of Genomic and Precision Medicine, Beijing Institute of Genomics, Chinese Academy of Sciences, Beijing 100101, China; Center for Excellence in Animal Evolution and Genetics, Chinese Academy of Sciences, Kunming 650223, China; University of Chinese Academy of Sciences, Beijing 100049, China; CAS Key Laboratory of Genomic and Precision Medicine, Beijing Institute of Genomics, Chinese Academy of Sciences, Beijing 100101, China; University of Chinese Academy of Sciences, Beijing 100049, China; CAS Key Laboratory of Genomic and Precision Medicine, Beijing Institute of Genomics, Chinese Academy of Sciences, Beijing 100101, China; University of Chinese Academy of Sciences, Beijing 100049, China; CAS Key Laboratory of Genomic and Precision Medicine, Beijing Institute of Genomics, Chinese Academy of Sciences, Beijing 100101, China; University of Chinese Academy of Sciences, Beijing 100049, China; CAS Key Laboratory of Genomic and Precision Medicine, Beijing Institute of Genomics, Chinese Academy of Sciences, Beijing 100101, China; CAS Key Laboratory of Genomic and Precision Medicine, Beijing Institute of Genomics, Chinese Academy of Sciences, Beijing 100101, China; CAS Key Laboratory of Genomic and Precision Medicine, Beijing Institute of Genomics, Chinese Academy of Sciences, Beijing 100101, China; Beijing Advanced Innovation Center for Big Data-Based Precision Medicine, School of Medicine and Engineering and Key Laboratory of Big Data-Based Precision Medicine (Ministry of Industry and Information Technology), Beihang University, Beijing 100191, China; State Key Laboratory of Genetic Resources and Evolution, Kunming Institute of Zoology, Chinese Academy of Sciences, Kunming 650223, China; Center for Excellence in Animal Evolution and Genetics, Chinese Academy of Sciences, Kunming 650223, China; University of Chinese Academy of Sciences, Beijing 100049, China; Key Laboratory of Animal Models and Human Disease Mechanisms of the Chinese Academy of Sciences and Yunnan Province, Kunming Institute of Zoology, Kunming 650223, China; CAS Key Laboratory of Genomic and Precision Medicine, Beijing Institute of Genomics, Chinese Academy of Sciences, Beijing 100101, China; Center for Excellence in Animal Evolution and Genetics, Chinese Academy of Sciences, Kunming 650223, China; University of Chinese Academy of Sciences, Beijing 100049, China; State Key Laboratory of Genetic Resources and Evolution, Kunming Institute of Zoology, Chinese Academy of Sciences, Kunming 650223, China; CAS Key Laboratory of Genomic and Precision Medicine, Beijing Institute of Genomics, Chinese Academy of Sciences, Beijing 100101, China; Center for Excellence in Animal Evolution and Genetics, Chinese Academy of Sciences, Kunming 650223, China; University of Chinese Academy of Sciences, Beijing 100049, China

**Keywords:** density-dependent selection, trade-offs, cancer cell, phenotypic diversity, competition

## Abstract

Classical *r-* vs. *K*-selection theory describes the trade-offs between high reproductive output and competitiveness and guides research in evolutionary ecology. While its impact has waned in the recent past, cancer evolution may rekindle it. Herein, we impose *r*- or *K*-selection on cancer cell lines to obtain strongly proliferative r cells and highly competitive K cells to test ideas on life-history strategy evolution. RNA-seq indicates that the trade-offs are associated with distinct expression of genes involved in the cell cycle, adhesion, apoptosis, and contact inhibition. Both empirical observations and simulations based on an ecological competition model show that the trade-off between cell proliferation and competitiveness can evolve adaptively. When the r and K cells are mixed, they exhibit strikingly different spatial and temporal distributions. Due to this niche separation, the fitness of the entire tumor increases. The contrasting selective pressure may operate in a realistic ecological setting of actual tumors.

## INTRODUCTION

Diverse environmental conditions act on populations and species, leading to selection-driven emergence of niche-specific adaptive phenotypes and preventing the emergence of a ‘superorganism’ [1]. Such a superorganism, often dubbed ‘Darwinian demon,’ would produce very large numbers of offspring and live indefinitely [2]. Existence of such entities is contrary to life history theory and empirical observation. Indeed, evolution of adaptive traits is typically restricted by fitness constrains [3]. These constrains often take the form of trade-offs whereby a life history trait can affect different components of fitness in opposite directions [4]. In contrast to natural organisms, cancers appear to be exempt from all constraints during the process of somatic cell evolution. A series of biological features, the so-called ‘hallmarks of cancer,’ are characterized by fast proliferation, resistance to low oxygen and crowded environment, and the ability to recruit blood vessels and escape the immune system [5]. How can all aspects of fitness be maximized in cancers? Perhaps heterogeneity within tumors enables several cell lineages to adopt a variety of characteristics and colonize different niches in a changing environment [6–14]. The internal and external microenvironments that cancer cells are confronted with in a multicellular organism are akin to complex ecosystems [15–21]. Trade-offs between cell proliferation and survival may apply to such cancer cell populations [6,22]. Both rapid cell proliferation and stable survival strategies must complement each other to achieve high fitness of a tumor as a whole [6]. Selection pressures that govern the trade-off between increasing proliferation and survival, and the ecological mechanisms that underlie these trade-offs in heterogenous populations remain uncertain.

A well-defined environmental variable governing evolutionary change is population density relative to essential resources [23]. The theory of density-dependent natural selection, often called *r*- and *K*-selection, states that at extreme population densities evolution produces alternative strategies [24]. The trade-offs are presumed to arise because the genotypes with the highest fitness at high population densities have low fitness at low density and vice-versa [3,25,26]. The *r*-populations are selected for high intrinsic rate of growth (r) in environments where population density is low and resources are abundant but perform badly at high density. In contrast, *K*-populations, experiencing strong competition for limited resources under high density conditions, should evolve high intraspecific competitive ability and enhance their carrying capacity (K). *K*-selected populations do not have high growth rates because they are near the carrying capacity for their environment [25,27].

**Figure 1.**
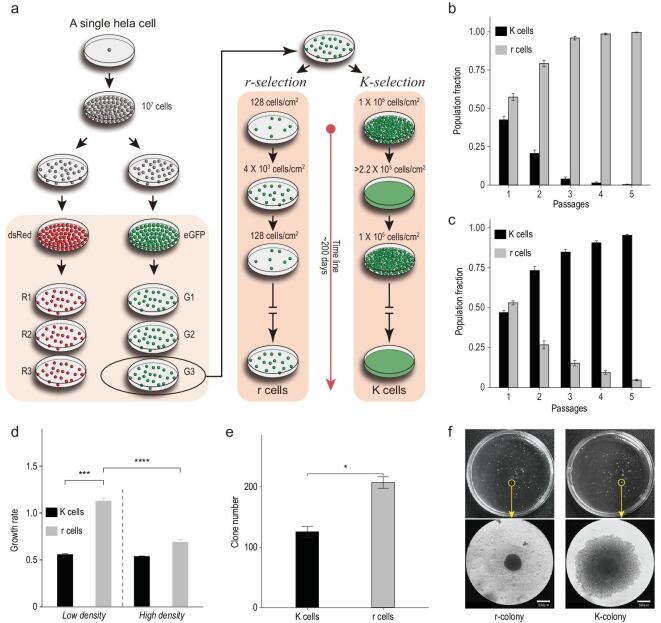
*r*- and *K*-selection in HeLa cells and their growth rate in 2D and 3D cultures. a) *r*- and *K*-selection strategies. An initial single cell clone was split into six populations, with three labeled with dsRed (R; red dots) and three with eGFP (G; green dots). Each cell line was cultured about 200 days at low (*r*-selection) and high density (*K*-selection). Fitness tests were performed at b) low and c) high density. The Y-axis is the proportion of r and K cells estimated by flow cytometry during five passages (x-axis) of r-K mixed cell cultures. d) The growth rate of r and K cells across culture conditions. Cells in 111 r- and 141 K-cell clones were counted every 24 hours. Growth rate is calculated based on cell number change within seven days. The tumorigenicity of r and K cells is presented based on the number e) and size f) of tumor colonies in a soft agar assay on the 7th and 21st day, respectively. The lower panels of f) present the microscopy images of one of the r and K colonies in the upper panels of f). Dash lines separate culture conditions or strategies. Error bars represent standard deviations. Student's *t*-test: ^*^P < 0.05, ^***^P < 0.001, ^****^P < 0.0001. Scale bars in f) represent 500 μm. *n* = 3 independent experiments per population in b), c), d), and e).

In this study, we performed artificial selection for cell density on HeLa cell line in order to amplify the diversity of cell growth within tumors (Fig. 1a). We asked whether selection under different density regimes modifies per capita growth rates and competitiveness as predicted by models that postulate a trade-off between *r*- and *K*-selection. To examine the phenotypic trade-offs at the molecular level, we carried out RNA-seq and explored the specific gene expression and pathway characteristics of r and K cells. The dynamics of density-dependent population growth in mixed populations change with the proportions of r and K cells within them. We modeled these dynamics and fitted our models to empirical observations in order to quantify the interaction among the various trade-off phenotypes in a heterogenous population and their effect on fitness of the entire tumor.

## RESULTS

### Fitness changes of *r*- and *K*-selected cells

The initial cell population (IN cells) was a single cell clone from a HeLa cell line. When the size of the population reached ${10^7}$ cells, we divided the clone into two sub-populations. One sub-population was marked with eGFP (IN_G) and the other with dsRed (IN_R) through lentivirus transfection. After approximately 200 generations under *r*-selection (the low-density condition) and about 130 generations under *K*-selection (the high-density condition), we obtained *r*-selected (r cells) and *K*-selected cells (K cells). The density-dependent selection scheme is illustrated in Fig. 1a.

To test whether r and K cells are more adapted to their corresponding conditions than the ancestral IN cells, we pairwisely co-cultured the three types of cells at high and low density. r cells become dominant within two passages (three days, Supplementary Fig. 1a) in the r-IN mix, suggesting that the r cells have evolved higher fitness than IN cells under these conditions. Likewise, K cells rapidly take over the K-IN mixed population (in four days, two passages, Supplementary Fig. 1b). Both r and K cells display better fitness than their counterpart in the r-K mix under corresponding selection conditions (Fig. 1b and c). We thus successfully selected for alternative life histories in our experiment.

### Trade-off between cell proliferation and survival

To explore the possibility that the r and K cells exhibit a trade-off in their density-dependent population growth, we first measured the growth rates of these cells in 2D *in vitro* systems at low and high density. Under low-density, r cells grow faster than K cells (Fig. 1d). When the test was performed at high density, there is no significant difference between r and K cells, whereas growth rates of r-cell populations decrease remarkably compared to low density conditions (Fig. 1d).

We next tested the difference between r and K cells in their density-dependent rates of population growth in 3D cellular environments. We quantified tumorigenicity by measuring colony growth and formation in a semi-solid agarose gel. The r cells displayed a significantly higher rate of colony formation than K cells within seven days (Fig. 1e). Afterwards, the number of colonies did not increase for both of r and K cells, while the colony size kept increasing. The K colonies grew faster than r colonies. Finally, K colonies were significantly larger than r colonies on day 21 (Fig. 1f). The diameter of K colonies was 0.46 mm (± 0.446) on average, while it was 0.31 mm (± 0.207) for r colonies. This suggests K cells have evolved to tolerate high density better than r cells.

**Figure 2.**
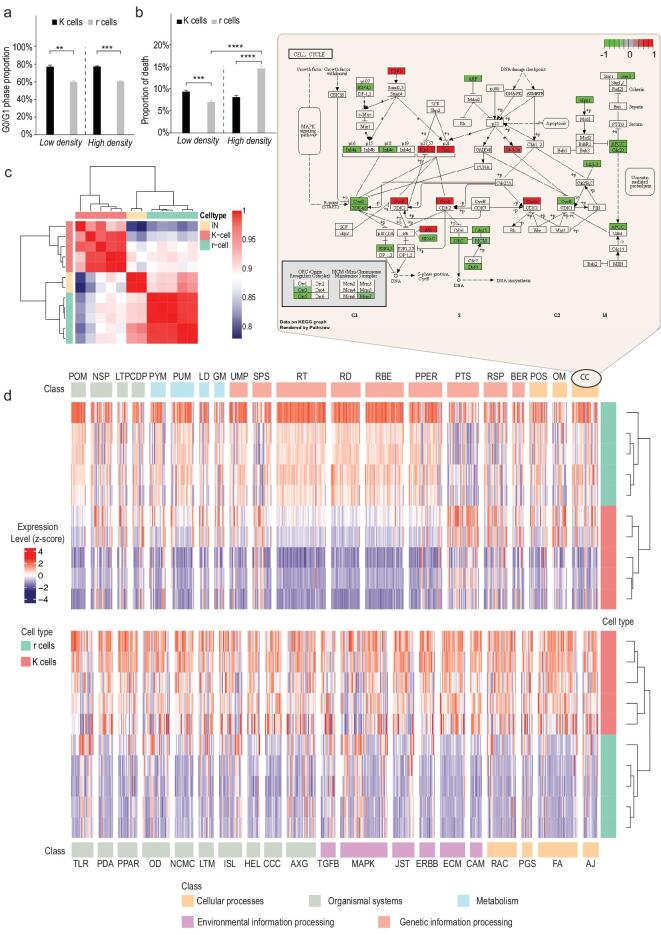
Differences in cell cycle, cell death and gene expression between r and K cells. a) The G0/G1 phase proportion and b) the proportion of cell death in r (gray) and K cells (black) are analyzed using PI and Annexin V staining via flow cytometry under high- and low-density conditions. Dashed lines separate culture conditions. Error bars represent standard deviations. (*n* = 3 independent experiments per population; Student's t-test: ^**^P < 0.01, ^***^P < 0.005, ^****^P < 0.0001; mean ± SD). c) Gene expression correlation between IN, r-, and K-cell populations. d) Pathways that show significantly different expression between r and K cells. The upper heatmap presents signaling pathways that are overexpressed in five K-cell populations (red), the bottom heatmap presents pathways overexpressed in five r-cell populations (green). The z-score heatmap indicates the scale of gene expression difference. The upper panel shows the cell cycle (CC; see Supplementary Table 6 for pathway abbreviations) pathway with relatively over- (red) and under- (green) expressed genes in r vs. K cells highlighted.

The net rate of population growth is determined by both cell death and birth rates. Using annexin-V and PI staining, reflecting cell death and the G0/G1 phase of the cell cycle, we measured the proportions of dead cells and distinguished the resting/quiescent (G0/G1) from total cells in the r and K populations at high and low density. Figure 2a shows that the proportion of G0/G1 phase cells is lower in the r*-* than in the K-cell populations, indicating that r cells proliferate relatively quickly at both low and high density. It also demonstrates that K cell birth rate does not increase at high density.

The K cell death rate is relatively stable under both conditions (Fig. 2b). In contrast, the r cell death rate increases significantly under high compared to low density. The r cells also die more frequently at high density than K cells (Fig. 2b). The high birth and death rates of r cells suggest that they have evolved to quickly produce offspring rather than to increase their survival, while K cells tend to ensure offspring quality rather than number. The high incidence of cell death leads to a decrease in growth rate of r cells at high density, and the effect of density in r*-*selected populations is mainly on cell death.

### Transcriptional divergence between r and K cells

To find molecular characteristics that may be correlated with the phenotypic trade-offs in r and K cells, we carried out RNA-seq in 22 samples, including two replicates of initial cell populations, five K cell lines, five r cell lines under routine cell culture conditions, and r- and K-replicate lines under high density stress. Multiple comparisons were performed among transcriptional profiles of cell lines across and within density conditions. Differentially expressed genes (DEGs) in these comparisons were identified using standard methods [28]. Figure 2c shows that r and IN cell populations cluster closely and differ from the K-cell populations under routine cell-culture conditions (at low density). We detect that 3161 genes show significant difference in gene expression between the r and K cells (Supplementary Table 1). Using the Functional Annotation Tool from the DAVID package [29], we found 25 pathways significantly enriched for these differentially expressed genes, including spliceosome, pathways involved in cancer, and ribosome biogenesis (Supplementary Table 2).

The top 20 highly expressed pathways in K or r cells based on the GAGE (General Applicable Gene-set Enrichment for Pathway Analysis) [30] are listed in Fig. 2d. The upregulated pathways in K cells include cell and focal adhesion, ErbB signaling, ECM-receptor interaction, phagosome, regulation of actin cytoskeleton, and Jak-STAT signaling. The cell cycle (upper panel in Fig. 2d), metabolism, and genetic information processing (such as ribosome biogenesis and mRNA surveillance) pathways are significantly highly expressed in r cells (Fig. 2d).

We next detected the transcriptional difference in responding to density constraints between r and K cells. Dramatic change at the transcriptional level is found in r cells when they are grown at high density. The expression levels of 6373 genes are significantly different from low density (Supplementary Table 1, Supplementary Data 2), while the number of DEGs is 2278 in K cells (Supplementary Table 1, Supplementary Data 3). Compared to the gene expression profiles under low-density conditions, 1775 genes present the same trend of expression change in both r and K cells under high density. These are involved in metabolic and serial RNA related pathways. These results suggest that high culture density has a prominent effect on cell metabolism (Supplementary Table 3). In addition to these common changes, only 503 ( = 2278−1775) genes respond to density change specifically in K cells. The number of genes (6373−1775 = 4598 genes) responding to the density change in r cells is approximately nine times larger than that, indicating that r cells are more sensitive and less stable at high density than K cells.

Previous studies found that the amount of expression plasticity between two environments is positively correlated with the fitness difference of the genotype between the two environments [31]. Changes in transcriptional profiles reveal that r cells are much more sensitive, in other words, less plastic to density change than K cells, consistent with the observation that r cells have lower fitness at high density in competition assays (Fig. 1b and c, Supplementary Fig. 1a and b). Differentially expressed genes that respond to density change in r cells are enriched in the cell cycle and DNA replication pathways (Supplementary Fig. 7 and 8), consistent with direct measurements of growth rate at high and low density (Fig. 1d).

### Suppression of contact inhibition pathways in K cells

The direct cellular response to cell density is contact inhibition which mediates cell growth and proliferation via interplay between growth signaling pathways and density constraints. Contact inhibition of proliferation is typically absent in cancer cells [32]. Both RNA-seq analysis and trypsinization assay showed that K cells are prone to form cell-cell adhesion at high density (Fig. 2d and Supplementary Fig. 6), implying a loss or decrease of contact inhibition [33]. In contrast, cell cycle arrest and the slower growth may still be triggered in r cells by signaling pathways that downregulate proliferation in a cell-density dependent manner [34]. One of such pathways, and well-studied, is the Hippo-YAP signaling pathway, which is largely responsible for inhibiting cell growth and controls organ size in many organisms [35]. The RNA_seq results in this study show that the Hippo signaling pathway is overrepresented in gene expression comparison between r and K cells, and expression of *YAP/TAZ* is significantly upregulated in K cells (Supplementary Data 1, Supplementary Table 2). In addition, the crosstalk among the Hippo signaling and eight other pathways (including adherens junction, focal adhesion, tight junction, PI3K-Akt signaling, mTOR signaling, ErbB signaling, TGF-beta signaling, and Wnt signaling) constructs a regulation network associated with cell cycle, cell survival, cell proliferation, and apoptosis [36]. A gene cluster analysis shows that the r and K cells can be distinguished by the expression profile of DEGs involved in these nine signaling pathways (Supplementary Fig. 2).

**Figure 3.**
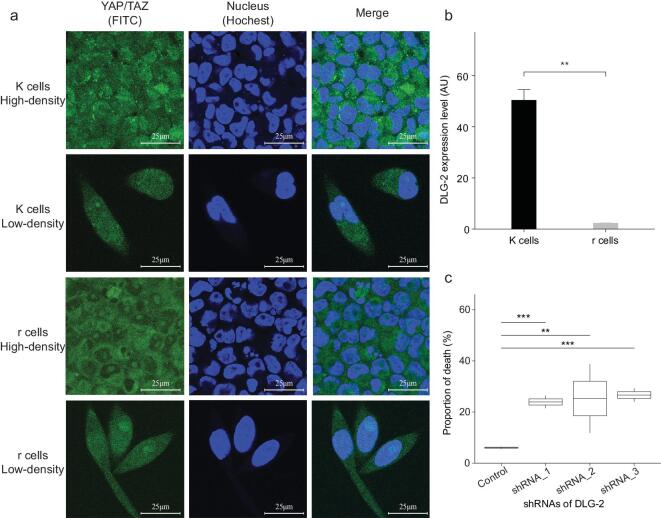
*YAP/TAZ* colocalization in r and K cells under high density and the effect of *DLG-2* knock-down in K cells. a) *YAP/TAZ* colocalization in the cytoplasm and nuclei under high density. *YAP/TAZ* was stained with FITC via immunofluorescent. Hoechst staining marks nuclei. Scale bars represent 25 μm. b) The expression level of *DLG-2* in r and K cells under high cultural conditions. The expression levels were validated by q-PCR. c) The proportion of cell death in *DLG-2* knockdown K cells under high density. The death rate was measured by Annexin V staining via flow cytometry. (Student's *t*-test: ^**^P < 0.01, ^***^P < 0.005; *n* = 8 independent experiments per population; mean ± SD).

The expression of anti-apoptotic factors can be activated by the transport of dephosphorylated *YAP* into the cell nucleus [37]. In reacting to high cell density, activated *LATS1/2* regulates phosphorylation of the coactivator *YAP/TAZ*, promoting cytoplasmic localization of *YAP* and leading to cell apoptosis and restriction of organ overgrowth. Overexpression or hyperactivation of *YAP/TAZ* has been observed in many types of tumors, stimulating growth and proliferation [38]. We performed an immunofluorescence assay to identify the localization of *YAP/TAZ* in r and K cells under both low- and high-density conditions. The localization of *YAP/TAZ* in the cytoplasm and nuclei was observed in both r and K cells at low density (Fig. 3). In contrast, the nuclear localization of *YAP/TAZ* is absent in r cells but is still maintained in K cells grown at high density (Fig. 3a). This suggests that *YAP/TAZ* phosphorylation is inhibited in K cells under high density, resulting in the loss of cell contact inhibition [39]. Consequently, cell apoptosis may be triggered by cytoplasmic localization of *YAP* in r cells but not in K cells as cell density increases.

In addition, *DLG-2* is a cell polarity gene in the hippo signaling pathway, regulating the inhibition of phosphorylated active *YAP/TAZ* proteins in the cytoplasm [40]. Our transcriptome analysis shows that expression of *DLG-2* is significantly higher in K cells at high than at low density (Supplementary Data 1). We confirmed this by RT-PCR (Fig. 3b). We carried out an siRNA assay to knock down the expression of *DLG-2* in K cells (Supplementary Fig. 3). The apoptosis rate of *DLG-2* knock-down K cells significantly increased at high density (Fig. 3c), confirming that the high expression level of *DLG-2* contributes to survival of K cells grown under these conditions.

### The competitiveness of r and K cells

The increased death rate of r cells and the underrepresentation of contact inhibition in K cells at high density indicate that cell interaction strength may be associated with the change in fitness of r and K cells as the environmental density increases. The theory of *r-* and *K-*selection predicts that populations living at high density and, hence, experiencing strong competition for limited resources should evolve high competitive ability [41]. As a result, the carrying capacity of *K*-selected populations is enhanced. In contrast, *r*-selected populations are typically far from their carrying capacity and thus can grow exponentially using an abundance of available resources. Competition among the members of an r population is supposed to be weak, which is disadvantageous for the fitness of these populations when space and resources are limited. The prediction implies that K cells should outcompete r cells in co-cultures under crowded conditions. In order to test this prediction, we established a co-culture assay of r and K cells to illustrate their temporal and spatial distribution. In addition, we simulated the dynamics of r and K mixed population distributions based on the Lotka–Volterra model [42,43]. Parameters of interaction strength between r and K cells in the simulations were estimated by fitting our models to empirical observations.

#### Empirical observations

**Figure 4.**
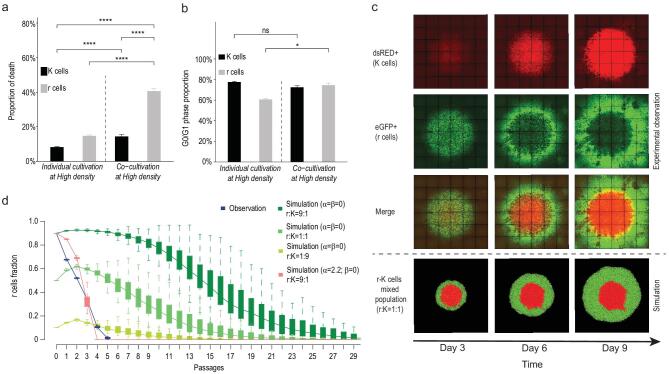
Inter-population interaction and temporal and spatial growth of r and K cells in mixed populations. a) Cell death and b) G0/G1 phase proportion of r and K cells in individual and mixed cultures. The y-axis in a) and b) shows death rates and G0/G1 phase proportion of r (gray) and K (black) cells. Death rates were measured by Annexin V staining. G0/G1 phase proportions were measured by PI staining via flow cytometry. Cells were cultured alone or co-cultivated at high density. Dashed lines separate culture strategies. (*n* = 3 independent experiments per population; Student's *t*-test: ns: non-significant, ^*^P < 0.05, ^****^P < 0.0001; mean ± SD) c) Spatial structure in an r-K mixed population. K and r cells are well mixed in equal proportion and seeded in the center of a six-well plate with total cell number ${\sim}{10^6}$. Each column represents time points from the 3^rd^ to 9^th^ day after cell seeding. r and K cells are eGFP and dsRed positive shown in green and red, respectively. The top and bottom panels show the spatial distribution of r and K cells in empirical observations and computer simulations, respectively. d) The distribution of *r* cell fractions estimated *in vitro* (blue (r:K = 9:1)) and *in silico* (red (α = 2.2, β = 0; r:K = 9:1); green (α = β = 0; r:K = 9:1); light green (α = β = 0; r:K = 1:1) and green yellow (α = β = 0; r:K = 1:9)). The y-axis reflects the fraction of *r* cells in the co-culture; x-axis represents cell passages. (*n* = 100 stochastic simulations per population; *n* = 3 independent experiments; mean ± SD).

Population proportion changes, as well birth and death rates of r and K cells were measured in a co-culture assay. When r and K cells are co-cultured at high density, the proportion of r cells decreases over time (Fig. 1c, Supplementary Fig. 4) and the death incidence of r cells is significantly higher than of K cells (Fig. 4a). The death rate and G0/G1

phase proportion among r cells in co-cultures are both significantly higher than when the r cells are cultured individually under crowded conditions (Fig. 4a and b). Compared to r, K cells have a relatively stable incidence of death and proportion of cells in G0/G1 phase under co-culture or in individual cultures, although their death rate increases under co-culture (Fig. 4a and b). These results show that the birth of r cells is restrained and cell death is accelerated when these two different types of cells are cultured together at high density, suggesting that they are in competition when they coexist.

Competition may result in niche separation among co-existing populations in an ecological community [44]. To examine this possibility, we carried out co-cultures where approximately 10^6^ r and K cells were well mixed at equal proportion and seeded in the centers of wells in six-well plates. Three replicate co-cultures were scanned every 72 hours. We observed that r and K cells in the co-culture assay tended to occupy different regions in a well. The r cells disperse to the periphery, while K cells grow and occupy the crowded central area (the upper panel of Fig. 4c). This observation reveals an additional density-dependent difference in the phenotypes of r and K cells.

#### Simulation and parameter estimation

To investigate the inter-population relationship between r and K cells, we adopted the Lotka-Volterra model which has been widely used to study population interaction [45,46]. We constructed computer simulations and looked at r and K cell population growth in co-cultures, and estimated the effect of K cells on r cells$\ ( \alpha )$, and vice versa ($\beta )$, respectively (equation (1b), see Methods). Parameter estimation equations are described in methods and materials.

Mixed populations were initiated in our computer simulations with different fractions of r and K cells (Materials and Methods), followed by 30 cell passages at high density. We compared the growth curves of r and K populations in the simulation to the empirical observations described in the previous section. Figure 4d shows that even when the initial proportion of r cells was lowest (r:K = 1:9) the extinction time of r cells in the simulation with no between-cell type interaction ($\alpha $ = $\beta $ = 0; no effect of one cell population on the other) is approximately five times longer than observed with the highest proportion of r cells (r:K = 9:1). Simulations reveal that the extinction time of the r cell population is shortened when α is higher than $\beta $ (Supplementary Fig. 5). Comparing the growth curves from empirical observations (blue line in Fig. 4d) and in simulations across values of $\alpha $ and$\ \beta $ (green and red lines in Fig. 4d), we find that the values of α = 2.2 and $\beta $ = 0 fit the data best (Fig. 4d and Supplementary Fig. 16). Thus, we infer that there is an interaction between r and K cells and K cells influence r cell death.

### Phenotypic diversity and competition promotes tumor fitness

#### In silico

**Figure 5.**
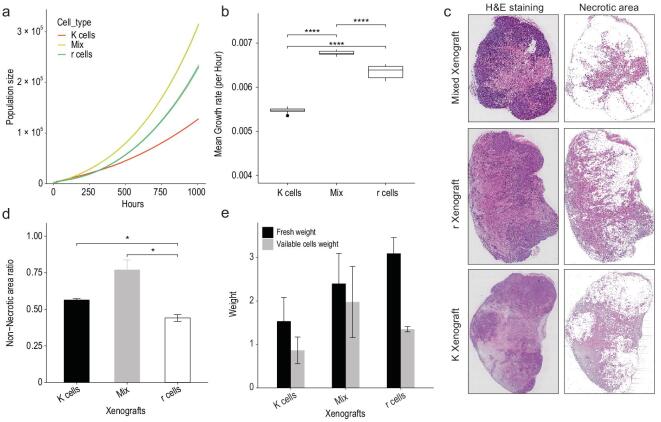
Population fitness of r, K*,* and r-K mixtures. a) Growth curves for different populations from the spatial computational model. The yellow line represents the r-cell population, the green line represents the mixture population of r and K cells and the red line represents the K-cell population. The Y-axis represents population size. The X-axis represents time. (*n* = 100 stochastic simulations per population; mean ± SD). b) Mean growth rate comparison among populations. The growth rate was measured at one-hour intervals. The Y-axis represents mean growth rate. The X-axis represents time. (*n* = 100 stochastic simulations per population; mean ± SD, Student's *t*-test: ^****^P < 0.0001). c) Necrotic area detection. The second column represents the necrotic area (colored) in xenografts. d) Proportion of the non-necrotic area (y-axis) in xenografts (*n* = 6 for each xenograft type; Student's *t*-test: ^*^P < 0.05, mean ± SD). e) Whole tumor (black) and viable cell (gray) weight in xenografts. The xenografts were extracted at the sixth week after cell inoculation. (*n* = 6 for each xenograft type; mean ± SD).

To test whether the existence of phenotypic diversity and inter-population interaction promote total fitness, we first carried out stochastic simulations to compare the growth dynamics of r-K mixed populations to pure r and K cells assemblages. Unlike in the previous section, the current computational model considers space and density heterogeneity in the environment where the tumor cells grow, and the interaction of r and K cells in these conditions. The rates of cell division and death depend on local cell density. Due to the density effect, cells are able to divide and migrate only if there is sufficient nearby space. The simulation is described in detail in the Materials and Methods and Supplementary Fig. 13. Figure 4c illustrates that *in silico* growth distribution of r and K cells in the mixed population is consistent with empirical observations (the upper panel of Fig. 4c). Among-cell interaction and the density effect promote the re-localization of r and K cells, from well-mixed at the beginning of cell culture to a biased distribution with the entire occupation of the K cells in the middle and the outward spread of r cells (the bottom panel of Fig. 4c). The mixed populations exhibit significantly higher rate of growth than the pure r- or K-cell populations (Fig. 5a and b).

#### In vivo

Mouse xenografts initiated with r, K, and r-K mixed cells were weighed on the 34^th^ day, followed by H & E staining. The necrotic and non-necrotic regions were distinguished using the gray threshold method [47]. We observed a high incidence of death in the r xenografts (Fig. 5c) and a significantly higher proportion of non-necrotic cells in the mixed xenografts (Fig. 5d). Although average fresh weight of the r xenografts is much larger than the fresh weight of K and mixed xenografts (reflecting the higher r cell proliferation rate, Fig. 5e), the mean weight of viable cells in the mixed xenografts is the highest. It indicates that the existence of phenotypic trade-offs within a cell population is advantageous to cell viability and population growth.

## DISCUSSION

*r*- and *K*-selection theory predicts that natural selection increases density-dependent rates of population growth. The notion of trade-offs in life-history evolution became a prominent feature of the theory and prompted a focus of theoreticians and field scientists both in ecology and evolutionary biology [3,24,27,48]. However, the heart of continuing controversy on the theory of *r*- and *K*-selection between theoreticians and field biologists is that many complex life-history characters of natural populations contradict theoretical expectations [27,49,50]. It is unrealistic to expect that a theory could account for all aspects of the natural environment and its impact on evolutionary processes in all organisms [27,49,51]. Thus, an optimal way to test the theoretical predictions is in controlled settings congruent with the assumptions of the simple models.

Tumorigenesis is an evolving and dynamic process where highly genetically and phenotypically heterogeneous neoplastic cell populations persist in challenging environments [52,53]. In fact hallmarks of cancer cannot be acquired in all cancer cells all the time [54]. An important cell-to-cell phenotypic variability is determined by several exterior and interior constrains [6]. For instance, environments in tumors are both stable (but crowded, hypoxic, and nutrient-poor) in the interior, and fluctuating in nutrients, space, and interaction between the components in the microenvironment at the edge of the tumors [55]. The consequences of somatic cell evolution under complex environmental pressures parallel ecological processes in nature, with inevitable survival-reproduction trade-offs because organisms have to allocate limited resources among several functions that affect fitness. Neoplastic cells may also be subject to evolutionary trade-offs with respect to resource allocation and growth constraints [6,22]. The mixture of biotypes that form cancer cell populations can be characterized by survival-proliferation trade-offs, and directly quantified in controlled environments *in vitro*. Carrying out experimental evolution under *r*- and *K*-selection in cancer cell lines, we observe that cancer cell populations face a survival-reproduction trade-off. The higher growth and death rates in r cells, compared to K cells (Figs 1d and 2b), indicates that r cells are selectively favored to allocate the majority of their resources to reproductive activities at the cost of their ability to propagate under crowded conditions, consistent with the central idea of the *r*- and *K*-selection theory [50]. Our analysis of pathway enrichment and expression of differentially expressed genes reflects phenotypic differences in cell proliferation, cell death, and adhesion between *r* and *K* cells *in vitro* and *in vivo*. Notably, our observation that r cells always locate at the peripheral in the co-culture of r-K mix is consistent with previous reports in HCC and breast carcinoma cases. Those histopathological images by Ki67 staining suggested that the cells replication is faster at the edge of the tumor [56,57].

The positive correlations between r and K have been reported, which indicating that the trade-offs are not the whole story [58,59]. The r-K correlation appears trade-up in low-quality environments [58]. In this study, 1775 genes present concordant changes in r and K cells at high density and are enriched in the processes of response to hypoxia, regulation of apoptotic process, regulation of mRNA stability, and so on, based on GO Term and KEGG enrichment analysis (Supplementary Table 4). Although the question whether those changes correspond to the trade-up or the short-term response to the high-density stress remains unclear, it would be interesting to test if the trade-up between cell proliferation and survival could emerge during long-term adaptive evolution under certain kinds of poor conditions in tumor microenvironment. Moreover, both trade-up and trade-off may be triggered in the tumors in which the variations in blood flow, immunoreaction, and drug treatment lead to the heterogeneous and fluctuant microenvironment.

Computer simulations which integrate of *r*- and *K*-selection theory predictions and parameters of inter-cell interaction based on Lotka–Volterra models illustrate temporal and spatial dynamics of population growth of heterogeneous cell populations following *r*- and *K*-strategies. The growth curves based on empirical data and mathematical models show that growth rates and fitness of *r-* and *K-*selected cells follow the logistic equations predicted by theory. As density increases, K cells dominate mixed cell populations. Our simulations, fitted to empirical data, establish a competitive relationship between phenotypically diverse cancer cells. It indicates that a tumor is not a ‘Darwinian demon’ *per se*, but is a mix of diverged cell populations. The populations with trade-off phenotypes are competing for space and different resources in the micro-ecosystem during the cancer progression. In the short term, the competition may decrease whole-population fitness, whereas, it triggers niche differentiation leading cell types to occupy different niches, thus maximizing the use of available resources in the ecosystem and leading to the emergence of resistance to environmental stress, such as drug treatment as well [19,44]. Therefore, the competitive interaction between tumor cells further improves the total fitness of a tumor in the long term. Our analyses of life-history trade-offs are pertinent to evolutionary ecology as well as cancer biology.

## MATERIALS AND METHODS

For detailed materials and methods, please see the supplementary data.

## Supplementary Material

nwaa124_Supplement_File

## References

[1] Silvertown J . Demons in Eden: The Paradox of Plant Diversity. University of Chicago Press, 2008.

[2] Law R . Optimal life histories under age-specific predation. Am Nat 1979; 114: 399–417.

[3] Stearns S . Trade-offs in life-history evolution. Funct Ecol 1989; 3: 259–68.

[4] Cain ML , Bowman WD, Hacker SD. Ecology. Sinauer, 2014.

[5] Hanahan D , Weinberg RA. Hallmarks of cancer: the next generation. Cell 2011; 144: 646–74. 21376230 10.1016/j.cell.2011.02.013

[6] Aktipis CA , Boddy AM, Gatenby RA et al. Life history trade-offs in cancer evolution. Nat Rev Cancer 2013; 13: 883–92. 24213474 10.1038/nrc3606 PMC4010142

[7] Wu C-I , Wang H-Y, Ling S et al. The ecology and evolution of cancer: the ultra-microevolutionary process. Annu Rev Genet 2016; 50: 347–69. 27686281 10.1146/annurev-genet-112414-054842

[8] Li C , Hou Y, Xu J et al. A direct test of selection in cell populations using the diversity in gene expression within tumors. Mol Biol Evol 2017; 34: 1730–42. 28369576 10.1093/molbev/msx115

[9] Roerink SF , Sasaki N, Lee-Six H et al. Intra-tumour diversification in colorectal cancer at the single-cell level. Nature 2018; 556: 457–62. 29643510 10.1038/s41586-018-0024-3

[10] Angelova M , Mlecnik B, Vasaturo A et al. Evolution of metastases in space and time under immune selection. Cell 2018; 175: 751–65. 30318143 10.1016/j.cell.2018.09.018

[11] Ling S , Hu Z, Yang Z et al. Extremely high genetic diversity in a single tumor points to prevalence of non-Darwinian cell evolution. Proc Natl Acad Sci USA 2015; 112: E6496–505. 26561581 10.1073/pnas.1519556112 PMC4664355

[12] Wang H-Y , Chen Y, Tong D et al. Is the evolution in tumors Darwinian or non-Darwinian? Natl Sci Rev 2017; 5: 15–7.

[13] Chen B , Shi Z, Chen Q et al. Tumorigenesis as the paradigm of quasi-neutral molecular evolution. Mol Biol Evol 2019; 36: 1430–41. 30912799 10.1093/molbev/msz075 PMC7967884

[14] Maley CC , Galipeau PC, Finley JC et al. Genetic clonal diversity predicts progression to esophageal adenocarcinoma. Nat Genet 2006; 38: 468–73. 16565718 10.1038/ng1768

[15] Nowak MA . Evolutionary Dynamics: Exploring the Equations of Life. Cambridge: Harvard University Press, 2006.

[16] Basanta D , Anderson ARA. Exploiting ecological principles to better understand cancer progression and treatment. Interface Focus 2013; 3, doi: 10.1098/rsfs.2013.0020. 10.1098/rsfs.2013.0020 PMC3915838 24511383

[17] Maley CC , Aktipis A, Graham TA et al. Classifying the evolutionary and ecological features of neoplasms. Nat Rev Cancer 2017; 17: 605–19. 28912577 10.1038/nrc.2017.69 PMC5811185

[18] Korolev KS , Xavier JB, Gore J. Turning ecology and evolution against cancer. Nat Rev Cancer 2014; 14: 371–80. 24739582 10.1038/nrc3712 PMC13213539

[19] Yang KR , Mooney SM, Zarif JC et al. Niche inheritance: a cooperative pathway to enhance cancer cell fitness through ecosystem engineering. J Cell Biochem 2014; 115: 1478–85. 24700698 10.1002/jcb.24813 PMC4143896

[20] Tabassum DP , Polyak K. Tumorigenesis: it takes a village. Nat Rev Cancer 2015; 15: 473–83. 26156638 10.1038/nrc3971

[21] Hu Y , Chen A, Zheng X et al. Ecological principle meets cancer treatment: treating children with acute myeloid leukemia with low-dose chemotherapy. Natl Sci Rev 2019; 6: 469–79. 10.1093/nsr/nwz006 PMC8291445 34691895

[22] Boddy AM , Huang W, Aktipis A. Life history trade-offs in tumors. Curr Pathobiol Rep 2018; 6: 201–7. 30595969 10.1007/s40139-018-0188-4 PMC6290708

[23] Mueller LD . Density-dependent population growth and natural selection in food-limited environments: the drosophila model. Am Nat 1988; 132: 786–809.

[24] MacArthur RH , Wilson EO. The Theory of Island Biogeography. Princeton University Press, 2001.

[25] Lansing E , Velicer GJ, Lenski RE. Evolutionary trade-offs under conditions of resource abundance and scarcity: experiments with bacteria. Ecology 1999; 80: 1168–79.

[26] Mueller LD . Theoretical and empirical examination of density-dependent selection. Annu Rev Ecol Syst 1997; 28: 269–88.

[27] Parry GD . The meanings of r- and K-selection. Oecologia 1981; 48: 260–4. 28309810 10.1007/BF00347974

[28] Li B , Dewey CN. RSEM: Accurate transcript quantification from RNA-Seq data with or without a reference genome. BMC Bioinformatics 2011; 12, doi: 10.1186/1471-2105-12-323. 10.1186/1471-2105-12-323 PMC3163565 21816040

[29] Huang DW , Sherman BT, Lempicki RA. Systematic and integrative analysis of large gene lists using DAVID bioinformatics resources. Nat Protoc 2008; 4: 44. 10.1038/nprot.2008.211 19131956

[30] Luo W , Friedman MS, Shedden K et al. GAGE: Generally applicable gene set enrichment for pathway analysis. BMC Bioinformatics 2009; 10: 1–17. 19473525 10.1186/1471-2105-10-161 PMC2696452

[31] Ho W-C , Zhang J. Evolutionary adaptations to new environments generally reverse plastic phenotypic changes. Nat Commun 2018; 9: 350. 29367589 10.1038/s41467-017-02724-5 PMC5783951

[32] Kim S , Chin K, Gray JW et al. A screen for genes that suppress loss of contact inhibition: identification of ING4 as a candidate tumor suppressor gene in human cancer. Proc Natl Acad Sci USA 2004; 101: 16251–6. 15528276 10.1073/pnas.0407158101 PMC528940

[33] Takai Y , Miyoshi J, Ikeda W et al. Nectins and nectin-like molecules: roles in contact inhibition of cell movement and proliferation. Nat Rev Mol Cell Biol 2008; 9: 603–15. 18648374 10.1038/nrm2457

[34] Gumbiner BM , Kim N-G. The Hippo-YAP signaling pathway and contact inhibition of growth. J Cell Sci 2014; 127: 709–17. 24532814 10.1242/jcs.140103 PMC3924201

[35] Halder G , Johnson RL. Hippo signaling: growth control and beyond. Development 2011; 138: 9–22. 21138973 10.1242/dev.045500 PMC2998162

[36] Ma X , Li W, Yu H et al. Bendless modulates JNK-mediated cell death and migration in Drosophila. Cell Death Differ 2014; 21: 407–15. 24162658 10.1038/cdd.2013.154 PMC3921588

[37] Yu FX , Zhao B, Panupinthu N et al. Regulation of the Hippo-YAP pathway by G-protein-coupled receptor signaling. Cell 2012; 150: 780–91. 22863277 10.1016/j.cell.2012.06.037 PMC3433174

[38] Ören M , Aylon Y. The Hippo Signaling Pathway and Cancer. Ören M, Aylon Y (eds.), New York: Springer Science & Business Media, 2014.

[39] Zhao B , Li L, Lu Q et al. Angiomotin is a novel Hippo pathway component that inhibits YAP oncoprotein. Genes Dev 2011; 25: 51–63. 21205866 10.1101/gad.2000111 PMC3012936

[40] Humbert PO , Grzeschik NA, Brumby AM et al. Control of tumourigenesis by the Scribble/Dlg/Lgl polarity module. Oncogene 2008; 27: 6888–907. 19029932 10.1038/onc.2008.341

[41] Titman D . Ecological competition between algae: experimental confirmation of resource-based competition theory. Science 1976; 192: 463–5. 17731084 10.1126/science.192.4238.463

[42] Lotka AJ . The growth of mixed populations: two species competing for a food supply. J Washingt Acad Sci 1932; 22: 461–9.

[43] Lotka J . Natural selection as a physical principle. Proc Natl Acad Sci USA 1922; 8: 151–4. 16576643 10.1073/pnas.8.6.151 PMC1085053

[44] Hardin G . The competitive exclusion principle. Science 1960; 131: 1292–7. 14399717 10.1126/science.131.3409.1292

[45] He X , Ni WM. Global dynamics of the Lotka–Volterra competition–diffusion system with equal amount of total resources, II. Calc Var Partial Differ Equ 2016; 55: 1–20.

[46] Muhammadhaji A , Teng Z, Rehim M. On a two species stochastic Lotka-Volterra competition system. J Dyn Control Syst 2015; 21: 495–511.

[47] Mehta S , Mercan E, Bartlett J et al. Learning to segment breast biopsy whole slide images. *2018 IEEE Winter Conference on Applications of Computer Vision (WACV)* 2018, 663–72.

[48] Pianka ER . On r- and K-selection. Am Nat 1970; 104: 592–7.

[49] Long T , Long G. The effects of r and K selection on components of variance for two quantitative traits. Genetics 1974; 76: 567–73. 4208860 10.1093/genetics/76.3.567 PMC1213086

[50] Reznick DN , Bryant M MJ, Bashey F. r- and k-selection revisited: the role of population regulation in life-history evolution special feature. Ecology 2002; 83: 1509–20.

[51] Wen H , Wang H-Y, He X et al. On the low reproducibility of cancer studies. Natl Sci Rev 2018; 5: 619–24. 31258951 10.1093/nsr/nwy021 PMC6599599

[52] Navin N , Kendall J, Troge J et al. Tumour evolution inferred by single-cell sequencing. Nature 2011; 472: 90–5. 21399628 10.1038/nature09807 PMC4504184

[53] McGranahan N , Swanton C. Clonal heterogeneity and tumor evolution: past, present, and the future. Cell 2017; 168: 613–28. 28187284 10.1016/j.cell.2017.01.018

[54] Floor SL , Dumont JE, Maenhaut C et al. Hallmarks of cancer: of all cancer cells, all the time? Trends Mol Med 2012; 18: 509–15. 22795735 10.1016/j.molmed.2012.06.005

[55] Marusyk A , Almendro V, Polyak K. Intra-tumour heterogeneity: a looking glass for cancer? Nat Rev Cancer 2012; 12: 323–34. 22513401 10.1038/nrc3261

[56] Waclaw B , Bozic I, Pittman ME et al. Spatial model predicts dispersal and cell turnover cause reduced intra-tumor heterogeneity. Nature 2015; 525: 261–7. 26308893 10.1038/nature14971 PMC4782800

[57] Lloyd MC , Cunningham JJ, Bui MM et al. Darwinian dynamics of intratumoral heterogeneity: not solely random mutations but also variable environmental selection forces. Cancer Res 2016; 76: 3136–44. 27009166 10.1158/0008-5472.CAN-15-2962 PMC5384728

[58] Wei X , Zhang J. Environment-dependent pleiotropic effects of mutations on the maximum growth rate r and carrying capacity K of population growth. PLoS Biol 2019; 17: e3000121. 30682014 10.1371/journal.pbio.3000121 PMC6364931

[59] Reding-Roman C , Hewlett M, Duxbury S et al. The unconstrained evolution of fast and efficient antibiotic-resistant bacterial genomes. Nat Ecol Evol 2017; 1: 50. 28812723 10.1038/s41559-016-0050

