# Supplementary material for: Variation in the life history strategy underlies functional diversity of tumors
Source: Natl Sci Rev. 2020 Jun 5;8(2):nwaa124. doi: 10.1093/nsr/nwaa124 (PMC8288455; doi:10.1093/nsr/nwaa124)
Supplement: nwaa124_Supplement_File [file nwaa124_supplement_file.zip › Supplementary data- Materials and Methods.docx]

# **Materials and Methods**

## Materials Availability

All HeLa strains generated in this study will be made available upon request to the Lead Contact. Further information and requests for resources may be directed to, and will be fulfilled by the Lead Contact, Xuemei Lu (xuemeilu@mail.kiz.ac.cn.).

## Experimental Model and Subject Details

### **Cell line**

The HeLa cell line was provided by the Cell Bank, Type Culture Collection Committee, Chinese Academy of Sciences. The test for mycoplasma contamination was negative. The HeLa-HPV18 single-nucleotide variants [60] were identified in the cell line (Supplementary Data 4). The HeLa cells were cultured in complete DMEM (Gibco) medium containing 10 per cent FBS (Gibco) and antibiotics (100 μg/mL streptomycin and 100 units/mL penicillin, Sigma-Aldrich) at 37 °C in an atmosphere of 5 per cent CO_2_.

### **Density-dependent selection**

**Evolution experimental system:** The initial cell population (IN-cells) derived from a single cell which was randomly selected from the HeLa cell line. When the population size of IN-cells reached ${10}^{7}$, it was randomly divided into two sub-populations of equal size. Each of sub-population was labeled with fluorescent proteins as described above. Density-dependent selection was performed on labeled cells.

**r-selection:** Cells were cultured under low-density. To ensure low density, cells were seeded on the surface of a 10 cm dish with approximately $128 cells/\mathrm{cm}^{2}$. Every 120 hours when the population density reached to about $4\times{10}^{3} cells/\mathrm{cm}^{2}$, a subset of cells was transferred to a new plate to keep a similar density as the original population ($128 cells/\mathrm{cm}^{2})$. Six replicates (three with dsRed and three with eGFP) were maintained in this manner for almost 200 cell generations (200 days). Samples from each population were cryopreserved in liquid nitrogen every 40 days.

***K*-selection:** Cells were cultured under high-density. To ensure high density, cells were seeded on the surface of a 10 cm dish with approximately ${10}^{5} cells/\mathrm{cm}^{2}$. Every 72 hours, when the population density reached to about $2.2\times{10}^{5} cells/\mathrm{cm}^{2}$, a subset of cells was transferred to a new plate to keep a similar density as the original population (${10}^{5} cells/\mathrm{cm}^{2})$. Six replicates (three with dsRed and three with eGFP) were maintained in this manner for almost 130 cell generations (200 days). Samples from each population were cryopreserved in liquid nitrogen every 40 generations.

### **In vivo tumor growth**

Female BALB/c Nude mice were purchased from the Beijing Vital River Laboratory Animal Technology Co., Ltd. Mice were 5–10 weeks of age for all experiments and kept in germ-free environments in the Institute of Zoology, Chinese Academy of Science.

Five-week female BALB/c Nude mice were assigned randomly into cages upon arrival. IN cells, r cells, and K cells were suspended in normal saline separately. 50 μL cell suspension ($2\times{10}^{5} Cells/\mu L$) was inoculated under the inguinal skin of the mice. For each type of cell inoculation, three mice were randomly selected and sacrificed every week from the third week after inoculation. Xenografts were collected for further analysis. Sample sizes were determined empirically (based on experience of other investigators who did similar assays). The experiments were not performed blind. All animal study protocols were reviewed and approved by the review boards of the Institute of Zoology Animal Care and Use Committee, Chinese Academy of Science (ethical approval reference number IOZ-20150061) and were conducted in accordance with the National Institutes of Health Guidelines for the Care and Use of Laboratory Animals. The maximal tumor diameter of 20 mm was permitted by Institute of Zoology Animal Care and Use Committee, Chinese Academy of Science. None of the experiments in this study exceeded this limit.

### **Model fitting**

Density dependent population dynamics can be predicted using a variety of mathematical models [61]. The logistic and Gompertz growth models are most frequently used [62,63]. To determine which mathematical model is suitable for us to predict cell population dynamics, we first obtained cell population dynamics data over eight days via the MTT cell proliferation assay, then fit population dynamics curves to three models: Logistic, Gompertiz, and Exponential (Supplementary Figure 9). We created a nonlinear model for cell population growth based on the data from the MTT assay (fitnlm function in Matlab). The adjusted *r*-squared value of the logistic growth curve is 0.856, the Gompertz – 0.828, and exponential – 0.739. This suggests that the logistic growth model fits the data best.

### **Modeling population growth dynamics of r and K cells in a mixed population**

We chose the Lotka-Volterra (L-V) model of interspecific competition to investigate growth dynamics of the r- and K-cell populations in their co-culture [43,64]. The general L-V model of interspecific competition is comprised of the following equations for population 1 and population 2, respectively:

$$\left\{ \begin{aligned} \frac{dx_{1}}{dt}=R_{01}x_{1}\left( 1-\frac{x_{1}+\alpha x_{2}}{C_{1}} \right) \\ \frac{dx_{2}}{dt}=R_{02}x_{2}\left( 1-\frac{x_{2}+\beta x_{1}}{C_{2}} \right) \end{aligned} \right., (1a)$$

where $x_{i}$is the size of the population i; $R_{0i}$ is the intrinsic per-capita growth rate, and $C_{i}$ is the carrying capacity. The competition coefficient, $\alpha$ or $\beta$, represents the effect that one species has on the other. Given the absent of the effects of interspecific competition ($\alpha=\beta=0)$, the two population are independent; the growth rate of one population only depends on the density of its own.

In this study, the difference is that the density of each population is contributed by the number of both r and K cells in the cell mixture when $\alpha=\beta=0$. Therefore, the growth rate of one population depends on the density of the whole population. In this circumstance, the equations can be modified:

$$\left\{ \begin{aligned} \frac{dx_{1}}{dt}=R_{01}x_{1}\left( 1-\frac{N_{t}+\alpha x_{2}}{C_{1}} \right) \\ \frac{dx_{2}}{dt}=R_{02}x_{2}\left( 1-\frac{N_{t}+\beta x_{1}}{C_{2}} \right) \end{aligned} \right., (1b)$$

where $N_{t}$ represents the total cell number at time $t,$ i.e $N_{t}=x_{1}+x_{2}.$ The population 1 is the r-cell population, and the population 2 is the K. When $\alpha$ < 1 the effect of population 2 on population 1 is less than the effect of population 1 on its own members. Conversely, when $\alpha$ > 1 the effect of population 2 on population 1 is greater than the effect of intra-population. The $\beta$ in the second equation is interpreted in the same way.

**Per-capita growth rate (**$\boldsymbol{R}_{\boldsymbol{0}}\boldsymbol{)}$ **estimation:** The intrinsic per-capita growth rate of every cell must be known at the beginning as the population growth model calculates the growth rate of every cell and its progenitors separately. It is an easy way to evaluate the intrinsic per-capita growth rate of every cell at the beginning via random sampling if the intrinsic per-capita growth rate distribution of a cell population is known. We isolated 141 single cells from the K-cell population and 100 single cells from the r*-*cell population and cultured them separately in wells of 96-well plates. Cells were counted every day for each clone over five days. The growth each cell over five days is considered exponential because cell density is very low.

Growth rate was estimated using the equation (2):

$$\frac{dN(t)}{dt}=rN\left( t \right), (2)$$

where $dN(t)$ is the total number of cells at time $t$, and $r$ is a constant. To obtain a linear function, cell numbers were converted to base-2 logarithms. The least-squares method (LSM) was used to fit the linear regression in which the slope (r) of the regression line estimates the growth rate.

We assume that mutations do not drastically affect the growth rate immediately. Therefore, $r$ is equivalent to the $R_{0}$ of the initial single cell. We assume that the growth rate of any given type of cell comes from a specified normal distribution. We estimate distribution parameters from empirical growth rates of 141 K cells (for K cell simulations) and from 111 r cells. (Supplementary Table 5).

The distribution of the intrinsic per-capita growth rate of a cell population is $R_{0}\sim Norm \left( \mu, \sigma^{2} \right)$ and lies within the interval $R_{0}\in(-\infty, +\infty)$*.* The parameters of the intrinsic per-capita growth rate distributions are estimated using the function ‘*normfit*’ in MATLAB. $DR_{0r}\sim Norm \left( 1.1832, {0.2441}^{2} \right)$ and $DR_{0K}\sim Norm \left( 0.6823, {0.3764}^{2} \right)$ are the fitted distributions.

**Carrying capacity (C) estimation:** Given the logistic cell population growth curve, carrying capacity can be estimated using the logistic growth function. We seeded r and K cells separately on six-well plates separately and assessed population size every 24 hours. The initial population size was $1.5\times{10}^{3}$cells. The population size of each population was calculated by Countstar BioMarine every day. Nine days of data were collected. The population size was used to fit the growth curve. The growth curve fitting was based on a logistic growth function. Parameters are shown in the legend of Supplementary Figure 10. Adjusted R^2^ of the r-cell growth curve estimation is 0.985, and 0.991 for K cells. The P-value of the r-cell growth curve is $2.21\times{10}^{-8}$ and $6.4\times{10}^{-9}$ for K-cell. The estimate of the carrying capacity is $239120 \mathrm{cells}/{\mathrm{cm}^{2}}$ for r cells and $228280 \mathrm{cells}/{\mathrm{cm}^{2}}$for K cells. (Supplementary Figure 10).

Since apoptotic cells could not be distinguished when the cell counting was performed each day, cell number may have been over-estimated. The severity of overestimation of r-cell number as cell density increases because these cells go into apoptosis at a high rater as conditions become crowded. It is necessary to correct the estimation of carrying capacity to eliminate the effect of apoptotic cells on cell count. The carrying capacity could be corrected by

$$C_{cor}=C_{est}\left( 1-r_{apo} \right), (3)$$

where the $C_{cor}$ represents the corrected carrying capacity, $C_{est}$ represents the estimated carrying capacity via curve fitting, $r_{apo}$ represents the r or K-cell apoptosis rate under high density (data were collected via FACS analysis of cell apoptosis).

The carrying capacity of r cells was estimated as $C_{r}=1.937\times{10}^{4} \mathrm{cells}/{\mathrm{cm}^{2}}$ and of K cells as $C_{K}=2.2216\times{10}^{4} \mathrm{cells}/{\mathrm{cm}^{2}}$.

$\boldsymbol{\alpha}$ **and** $\boldsymbol{\beta}$ **estimation:** We simulated the dynamics of r and K mixed population with different pairwise of $\alpha$ and $\beta$ values using the equation (2). First, we used a grid-search scheme, all parameter pairs traversed with intervals 0.5, to estimate the $\alpha$ and $\beta$. Other parameters were fixed. We then calculated Pearson correlation coefficients between predictions and observations. These correlations are maximized when ($\alpha$, $\beta$) = (2.5,0) (Supplementary Figure 16). The values of Pearson correlation coefficients increase within the interval of $\alpha$ ∈ [2,2.5], and decrease within the interval of $\alpha$ ∈ [2.5,3] when $\beta$ =0. This suggests that the values of $\alpha$ between 2 to 3 and $\beta$ = 0 maximize the agreement between simulations and data. We next repeated the grid-search scheme, traversing values of $\alpha$ between 2 and 3 with intervals of 0.1 and setting $\beta$ = 0. The final estimates are: $\alpha$ = 2.2 and $\beta$ = 0 (Supplementary Figure 5, 11 and 16).

Cell population dynamics of the mixed population are based on the values of $C_{r}$,$C_{K}$, $\frac{C_{r}}{\alpha}$ and $\frac{C_{K}}{\beta}$. We estimate $C_{r}$ = $1.937\times{10}^{4} \mathrm{cells}/{\mathrm{cm}^{2}}$,$C_{K}$ = $2.2216\times{10}^{4} \mathrm{cells}/{\mathrm{cm}^{2}}$, $\alpha$ = 2.2, and $\beta$ = 0. Thus, $\frac{C_{r}}{\alpha}$ = 9.685$\times{10}^{3}$ and $\frac{C_{K}}{\beta}$ is infinite. Here $C_{K}>\frac{C_{r}}{\alpha}$ and $C_{r}<\frac{C_{K}}{\beta}$ indicate that r cells would eventually go extinct when competing with K cells [65].

### **Spatial growth model**

Tumor cells living in a limited space cannot move freely. Among-cell interactions are also confined to a limited space, precluding interaction when between-cell distance is large. Given these considerations, we assume that there is a cell-centric limited space for every cell where the density-dependent effects which impact the central cell are confined. We call this density-dependent space (DDS; for more details see Stochastic simulation of cell growth with spatial structure).

When a population grows logistically, its growth is exponential early on, provided it carrying capacity is much greater than its size. However, if carrying capacity is small, early-stage population size increase results in a drastic decrease in growth rate. Carrying capacity is related to the size of the habitat. In a DDS, the maximum carrying capacity is 36 (Supplementary Figure 13). Here we use $C_{s}$ to represent the carrying capacity in a DDS. Because the $C_{s}$ value is very small (compare to $C$ in equations (1b)), the equations (1b) are not applicable to predict the dynamics of r and K cells mixed population using the spatial growth model. In addition, the typic growth curve for cells grown in culture is a logistic curve in which $\frac{dN}{dt}$ reaches the maximum when $N=\frac{C}{2}$ during the logistic cell growth. In the DDS, only when cell density exceeds a certain threshold, do the cells become subject to density-dependent growth.

For two cell sub-populations, we let

$$X_{1}=N_{t}+\alpha x_{2}, (4)$$

$$X_{2}=N_{t}+\beta x_{1}, (5)$$

where $X_{1}$ and $X_{2}$ represent the practical population size which determines the density-dependent growth rate of population 1 and population 2, respectively.

In the DDS, the effects of heterogeneity of spatial density on cell growth rate are taken into consideration. We use two-interval piecewise function to express the function of $\frac{dx}{dt}$ for each of population 1 and 2. When cells are under low density condition where $X_{1}\leq\frac{C_{s1}}{2}$ or $X_{2}\leq\frac{C_{s2}}{2}$, the cell growth is exponential, and the growth rate is equal to the intrinsic growth rate of the cell. Given interspecific competition happens at high density, L-V model is applied when $X_{1}>\frac{C_{s1}}{2}$ or $X_{2}>\frac{C_{s2}}{2}$. Therefore, we have

$$\left\{ \begin{aligned} \frac{dx_{1}}{dt}=R_{01}x_{1}, if X_{1}\leq\frac{C_{s1}}{2} \\ \frac{dx_{1}}{dt}=R_{01}x_{1}\left( 1-\frac{2(N_{t}+\alpha x_{2})-C_{s1}}{C_{s1}} \right), if X_{1}>\frac{C_{s1}}{2} \\ \frac{dx_{2}}{dt}=R_{02}x_{2}, if X_{2}\leq\frac{C_{s2}}{2} \\ \frac{dx_{2}}{dt}=R_{02}x_{2}\left( 1-\frac{2(N_{t}+\beta x_{1})-C_{s2}}{C_{s2}} \right), if X_{2}>\frac{C_{s2}}{2} \end{aligned} \right., (6)$$

the coefficients in equation 6 are set such that the $\frac{dx_{1}}{dt}$ and $\frac{dx_{2}}{dt}$ are continuous at the $X_{1}=\frac{C_{s1}}{2}$ and $X_{2}=\frac{C_{s2}}{2}$ for our piecewise functions.

**Carrying capacity estimation of the spatial growth model:** In the spatial model, we assume that the density-dependent space (DDS) is a square area which contains 36 grids coordinates (6$\times$6 grids, Extended Date Figure 13). Because the carrying capacity of K cells is 1.147 times that of r cells ($\frac{C_{K}}{C_{r}}\approx1.147$; more details see the carrying capacity estimation), in a DDS the carrying capacity of K cells is $C_{sK}=36$ (the maximum number for the region), and of r cells is $C_{sr}=31$.

### **Stochastic simulation of population growth of r and K cells in co-cultures**

Cells in culture are subject to artifacts, such as subculture. A subculture is performed when cell density exceeds a threshold (roughly 70 per cent to 90 per cent confluent) and is used to maintain cell density. The subculturing procedure includes recommended split-ratios and cultural medium replenishment schedules. A realistic *in silico* cell culture model should take into account such artifacts. The details of the stochastic simulation procedures are as follows:

**Initiation:** We assign the initial intrinsic per-capita growth rate to every cell on initialization. Here we assumed that growth rates of cells in a population come from a normal distribution. Every cell is assigned an initial growth rate sampled from its growth rate distribution (see the per-capita growth rate estimation for details). To avoid outliers, random sampling was based on a truncated distribution (within the interval $R_{0}\in(Q1, Q3))$ of the intrinsic per-capita growth rate. $Q1$ is the lower quartile and $Q3$the upper quartile of the observed growth rate distribution respectively (Supplementary Table 5). The initial population size was chosen according to culturing methods being simulated and followed experimental conditions.

**Population growth and sub-culture:** Cell division is based on growth rate. Each cell in a population, enters the division stage only if the cell growth rate is over 0. In the stochastic simulation, the time of a cell cycle (CT) was defined as

$$CT={24}/R, (8)$$

where the $R$ represents the growth rate of a cell calculated from the density-dependent population growth model. CT is measured in hours. The biological meaning of $R$ is the number of cell divisions within 24 hours. Considering the characteristics of the cell cycle, the time of interphase of mitosis occupies nearly 90 per cent of the entire cycle [66]. Thus, cell division time (DT) is:

$$DT=0.9\cdot CT+geornd\left( 1/\left( 0.1\cdot CT \right) \right), (9)$$

where $geornd$ means a random number sampled from a geometric distribution. If the time since the last cell division is greater than DT, the cell divides into two cells. When the culture time exceeds 72 hours (*K*-selection conditions), subculture was performed. Population size was reduced to the initial population size during subculture by random selection. After subculture, cells continue to grow *in silico*. For the program structure, see Algorithm 1.

Six mixed populations with different r- and K-cell initial proportions (r:K=99:1, r:K =9:1, r:K =7:3, r:K =1:1, r:K =3:7, r:K =1:9) were simulated. 100 simulations were performed for each population type (Supplementary Figure 12).

### **Stochastic simulation of population growth with spatial structure**

Tumor cells live in a spacially heterogeneous environment [67,68]. The distribution of cell density within a tumor should therefore be non-uniform. Spatial structure plays an important role in population dynamics [56,69–73]. In a given cell growth space, if the real-time location of cells can be determined, the spatial structure of the whole population can be described. For this reason, we constructed a two-dimensional lattice-based growth space where physiological activities such as cell growth and migration are carried out. The location of cells is determined by grid coordinates. (Supplementary Figure 13)

To simulate the population dynamics of cells which grow on a two-dimensional surface as realistically as possible, we considered the following factors that can influence spatial structure: cellular morphological characteristics, cell migration, cell proliferation, and cell death.

**In-silico cellular morphology:** The growth of cells on a two-dimensional surface may result in regional differences in cell density due to uneven cell distribution or different growth rates. In other words, the density-dependent spatial heterogeneity exists in the cell growth environment. In addition, space occupied by cells varies under different densities. In a low-density environment, cells occupy a larger area, increasing cell surface to maximize contact with the culture medium. Because of crowding, cells are arranged closely. The attachment area of a cell decreases in a high-density environment (Supplementary Figure 14). Therefore, we assume cells have two in-silico morphological types: large cell, corresponding to cells growing in a low density environment, occupying four coordinate grid; small cell, corresponding to cells growing in high density environment, occupying one coordinate grid (Supplementary Figure 13). The *in silico* cellular morphology can be transformed between large and small cells. When there is an empty coordinate around a small cell that can accommodate a large cell, the small cell preferentially transforms into a large cell. A large cell will switch its morphology to two small cells via cell division when and only when there is no space around it that can accommodate two large cells, and there is space to accommodate two small cells.

**Cell migration:** Cells migrate with a certain rate in their growth space. When a migration event occurs, the coordinates of cells in the growth space change once, and the migration must occur to adjacent coordinates (Supplementary Figure 13). The migration direction of each step is assumed random. Cells differ in their migration speed. *r* cells migrate more readily than K cells, as measured by a trans-well migration assay. The mean migration speed of r cells is close to five times higher than K cells (Supplementary Figure 15). Here we assume that the migration speed follows a beta distribution. The parameter “a” of the beta distribution is 5. The expected migration speed of r cells is 0.5 and of K cells is 0.1.

**Cell proliferation:** A division event can only be completed in two adjacent coordinates. Cells retain their original cellular morphology during division. If there is no space to proliferate, small cells die.

**Cell death:** When a cell dies, its original coordinate is marked as empty and can be occupied by another cell via cell division or migration. Death occurs if a cell that must divide but has no space to do so, or if a cell is affected by high density (calculated by Equations (7)).

**Density-dependent space:** Density-dependent space (DDS) is a square region containing 36 grids (6$\times$6 grids). A cell can be in the center (large cells) or on the grid coordinate (3,3) whose origin is the top-left of the DDS grid (small cells). We assume that only the cells located in the DDS contribute the density effects to the central cell (Supplementary Figure 13).

**Simulation process:** The simulation program of cell growth with spatial structure is divided into two processes: initiation and population growth. In the initiation process, cells were loaded in the center of the growth space. All cells were clustered together to form a circle community. This constructs a density-dependent spatially heterogeneous environment for cell growth. The outside space low-density, while inside the cell community is a relatively high-density environment. The intrinsic per-capita growth rate (Normal distribution; the same as the initiation process in the stochastic simulation of cell growth with subculture) and migration speed (Beta distribution) of each cell were initiated with random sampling. Finally, the program calculates a constant variable δt, which represents the minimal time interval that can contain a migration event. In the population growth process, the migration and proliferation of cells depend on the migration rate and the density growth rate (calculated by Equations (7)). The migration rate and the intrinsic per-capita growth rate were maintained between mother and daughter cells. The program iterates all cells and calculates their density growth rates. δt is the time interval between iterations. For the program structure, please see Algorithm 2.

## Algorithm 1

Outline description of the algorithm used to implement the Stochastic simulation of cell growth with subculture. The program was written in C++.

Parameters calculation;

Create initial population;

Passages = 0;

Define δt;

***while*** Passages < Passages_limit ***do***

Calculate the r-cell and K-cell number;

***for*** each cell *i* ***do***

Calculate the expected growth rate; (Equations (1b))

***end***

t = 0;

***while*** t< t_limit ***do***

calculate the r-cell and K-cell number;

***for*** each cell *i* ***do***

Cell death judgements;

Calculate the expected growth rate; (Equations (1b))

***end***

Eliminate the dead cells;

Recalculate the r-cell and K-cell number;

***for*** each cell *i* ***do***

***if*** t = Cycling time ***then***

Division;

Calculate the expected growth rate; (Equations (1b))

***end***

***end***

Increase time t by δt;

***end***

Passage;

Record subpopulation fraction;

Increase Passages by 1;

**end**

## Algorithm 2

Outline description of the algorithm used to implement the Stochastic simulation of cell growth with spatial structure. The program was written in C.

Parameters calculation;

Create initial population;

Calculate δt;

time = 0;

***while*** t< t_limit ***do***

***for*** each cell *i* ***do***

Calculate the r-cell and K-cell number in a density dependent region;

Calculate the expected growth rate; (Equations (6))

***end***

***for*** each cell *i* ***do***

Cell death judgements;

Calculate the expected growth rate; (Equations (6))

***end***

Eliminate the dead cells;

Recalculate the r-cell and K-cell number in a density dependent region;

***for*** each cell *i* ***do***

***if*** t = Cycling time ***then***

Division;

***end***

***elseif*** t = migrating time ***then***

Migration;

***end***

***end***

Increase time t by δt;

***end***

## Method Details

### **Cell cryopreservation**

Cells were first trypsinized using a 1X trypsin-EDTA solution at room temperature for three minutes and suspended in complete growth medium. Suspended cells were collected by centrifugation (1300 rpm, 5 min) and resuspended in 1X PBS. PBS suspended cells were collected by centrifugation (1300 rpm, 5 min) and resuspended in cryopreservation medium. The cryopreservation medium contains 10 per cent DMSO and 90 per cent FBS. Cryopreservation medium suspended cells were pipetted into a cryopreservation vial gently, and placed into a -80 °C freezer. Finally, vials were transferred intoliquid nitrogen for long-term storage when temperature decreased to -40°C.

### **Subculture and Single-cell isolation**

Cells were washed with 1X PBS three times after discarding cell culture medium, and trypsinized with 1X trypsin-EDTA solution at room temperature for three minutes. The detached cells were suspended, divided, and transferred into plates. Single cells were sorted into individual wells of 96-well plates by flow cytometry (BD) from a HeLa cell population. After six hours, a microscopic examination was performed to ensure only one cell in a well.

### **eGFP and dsRed transfection**

Cells were transfected by Lentiviral vectors pLenti6.3-MCS-IRES-eGFP and pLenti6.3-MCS-IRES-dsRed (Invitrogen). Approximately $5\times{10}^{6}$ HeLa cells were incubated in a 10cm dish with DMEM before transfection. After incubating for 24 h, the DMEM medium was replaced by 10 mL transfection-mix-medium that contains 8 μg/mL polybrene and ${10}^{6}$ IU/mL lentivirus particles. The multiplicity of infection (MOI) value was 1. After transfecting for 24 hours, cells were washed with PBS three times. To select cells that stably express eGFP and DsRed, the transfected cells were cultured in DMEM medium with blasticidin (10 µg/mL) for at least four weeks.

### **Relative fitness assay**

To measure the relative fitness of two cell populations cultured under a specific cell density (routine-, r-, or K-), the two cell populations were mixed and cultured together. The proportions of the two populations were monitored by flow cytometry (BD, Ex/Em (nm): 346/442) once a subculture was performed. Time between two subcultures depended on the culture protocol. The higher fitness population is the one dominating the mixed population over time.

### **Measurement of growth rate**

Population growth rate was estimated using the equation (10):

$$\frac{dN(t)}{dt}=rN\left( t \right), (10)$$

where $dN(t)$ is the total number of cells at time $t$, and $r$ is a constant coefficient. To obtain a linear function, cell numbers were converted to base-2 logarithms. The least-squares method (LSM) was used to fit the linear regression with the slope (r) of the regression line estimating the growth rate.

### **Soft agar colony formation assay**

Approximately 1,000 cells were suspended in a top layer of 0.4 per cent soft agar (SeaPlaque Agarose, BMA products). The cell suspensions were then overlaid onto a bottom layer of 1 per cent soft agar containing complete DMEM supplemented with 10 per cent FBS in six-well plates. After a week, colony number was counted. After three weeks, the images of colonies were collected to compare their diameters by microscopy.

### **FACS analysis of G0/G1 phase and cell death/apoptosis**

Cells were trypsinized and suspended in cold 1X PBS.Five μL propidium iodide (Sigma, P4170) was added to the suspension. Cells were incubated at 4 ºC for 30 minutes. Stained cells were analyzed by flow cytometry (BD, Ex/Em (nm): 346/442). Data were collected from 10000 stained cells.

The Annexin V, Alexa Fluor® 350 conjugate (Invitrogen, a23202) was used for apoptosis rate detection. Cells were trypsinized and diluted to ~1 $\times$ 10^6^ cells/mL in the annexin binding buffer. 5 μL annexin V conjugate was added to 100 μL of the cell suspension. The cell suspension was incubated at room temperature for 15 minutes. After the incubation, 400 µL annexin-binding buffer was added. The samples were kept on ice after mixing gently. The stained cells were analyzed by flow cytometry (BD, Ex/Em (nm): 346/442). Data were collected from 10000 stained cells.

### **Necrotic area detection and calculation of the weight of viable cells in mouse xenografts**

The H&E (haematoxylin and eosin) staining of tumor sections was used to detect necrotic cells[74]. First, we prepared the central H&E staining section of a xenograft. The sections were then converted into digital images using Aperio Digital Pathology Slide Scanner (Leica). To detect necrotic areas in a xenograft, the images of sections were read via Matlab (MathWorks) and converted to gray scale (rgb2gray function in Matlab). Image contrast was enhanced using histogram equalization (histeq function in Matlab). We then adjusted image intensity values twice with parameters low_in=0.1 and high_in=0.7 (imadjust function in Matlab) and applied the 2-D median filtering to the image with the filtering parameters m=5 and n=5 (medfilt2 function in Matlab). Finally, we set grey scale value 90 as the threshold to distinguish the necrotic and non-necrotic areas of the image. The pixels in the tumor region with the grey scale value less than 90 were considered necrotic. The net weight of viable cells in a xenograft tumor was obtained by multiplying the total weight of a tumor by the proportion of the non-necrotic area.

### **Trypsinization assay**

Cells were seeded on a six-well plate. After 12 hours, we discarded the culture medium and washed the cells with cold PBS three times. 500µL 0.05 per cent Trypsin was added into the well at room temperature. The plate was swayed softly and slowly 20 times. All supernatant (about 500 µL) was transferred into a new tube. We pipetted the supernatant gently to make sure most cells were single individuals. 400 µL of the supernatant was put back on the plate to trypsinize the remaining cells. Finally, we estimated cell numbers in the 100 µL of the remaining supernatant ($N_{1}$) and among the remaining cells ($N_{2}$) using a hemocytometer. The following equation was used to calculate the trypsinised cell ratio within a time interval:

$$Ratio= \frac{N_{1}}{N_{1}+N_{2}} \times100 per cent. (11)$$

### **Immunofluorescence assay**

Cells were seeded on coverslips. The coverslips were then placed on six-well plates. After a 48-hour incubation, cells were fixed with 4 per cent paraformaldehyde (PFA) in PBS for 20 minutes at room temperature, followed by blocking and permeabilizatuion for 30 minutes in blocking buffer, comprising 2 per cent bovine serum albumin (BSA) and 0.2 per cent Triton X-100 in PBS. Cells were incubated with the Yap1 antibody (GTX35195, GeneTex) for one hour, then with the FITC-conjugated goat anti-rabbit IgG (H+L) polyclonal antibody (GTX77059, GeneTex) for 30 minutes. Both antibodies were diluted in PBS with 2 per cent BSA. Cell nuclei were stained with Hoechst 33342 (H3570, Invitrogen). Images were acquired using a Leica TCS SP8 confocal laser microscopy system (Leica Microsystems).

### **Real-time quantitative PCR with reverse transcription**

Total RNA was isolated using the TRIzol reagent, as described by the manufacturer (15596018, Invitrogen). 1 μg of RNA was used to generate cDNA with the High Capacity cDNA Reverse Transcription Kit (4368814, Applied Biosystems). Real-time quantitative PCR was performed to amplify cDNA by using Maxima STBR Green/ROX qPCR Master (K0223, Thermo Scientific) in a CFX96 Touch Real-Time PCR Detection System (Bio-Rad). The average threshold cycle (Ct) of quadruplicate reactions was determined and amplification was analyzed by the ΔΔCt method. Gene expression was normalized to that of *GAPDH*. Real-time quantitative PCR with reverse transcription data were representative of at least three independent experiments, with two technical replicates per experiment. Primer sequences used to amplify human *DLG-2* and *GAPDH* as follows:

human *DLG-2* forward: CAATGGGATGGCAGACTTTT;

human *DLG-2* reverse: ACAGCTCGGTGGAGAAACAT;

human *GAPDH* forward: ACAGCCTCAAGATCATCAGC;

human *GAPDH* reverse: ATGGACTGTGGTCATGAGTC.

### **siRNA knockdown**

siRNAs (Lipofectamine 3000 transfection reagent) were used to knock down the expression of *DLG-2*. To check the knockdown efficiency, total RNA was isolated and quantified by quantitative PCR (qPCR) three days after transfection. The target sequences used to knock down human *DLG-2* are as follows:

si-h-DLG2_001: ACCUCAUUCUUUCCUAUGA;

si-h-DLG2_002: GCUAGAACAAGAAUUUGGA;

si-h-DLG2_003: GGAGAUGAAUAAGCGUCUA.

### **r/K competition assay in vitro**

K cells with dsRed and r cells with eGFP were mixed together equally. Cell density of the mixture population was about 2$\times$10^6^ cells/mL. 500 μL of cell mixture was loaded on the central surface of an empty culture plate. After five minutes, the plate was put back to the incubator. When all cells attached (almost two hours), sufficient complete growth medium was added to the plate. Microscopic fluorescent field images of the plate were collected using imageXpress XLS (Molecular Devices) every three days. Image data were analyzed following the pipeline in the imageXpress XLS data analysis software (Molecular Devices).

### **MTT assay**

Cells were suspended and seeded at the concentration of 500 cells/well in 96-well plate. A volume of 20 μL dissolved MTT was pipetted into each well. After incubating for 4 h at 37 °C in a humidified CO_2_ incubator, the medium was removed and 200 μL sterile DMSO was added to each well. Absorbance values were then read at 570 nm with a microplate spectrophotometer. The proliferation of living cells was monitored based on absorbance values.

### **Migration assay**

Migration assay was performed using 6.5mm Transwell inserts (3422, Corning) containing polycarbonate membrane filters (8-μm pore size) for 24-well plates. Briefly, cells were digested with 0.05 per cent trypsin, and suspended in an FBS-free DMEM culture medium. Cells were then plated into the upper chamber (3$\times$10^3^ cells/well). At the same time, 650 µL of DMEM with 10 per cent FBS was added to the lower chamber of the well and the plates were incubated for five hours at 37 °C with 5 per cent CO_2_. After incubation, cells on the upper surface of the membranes were removed gently with cotton swabs. Cells that had entered the lower surface of the filter membrane were stained with 0.1 per cent Hoechst 33342 (H3570, Invitrogen) for 30 minutes at room temperature, and washed three times with PBS. Four randomly selected fields in each well were image captured with the ImageXpress Micro HCS (Molecular Devices), and migrated cells were counted. n = 3 independent experiments.

## Quantification and Statistical Analysis

### **RNA-seq and data analysis**

Total RNA was isolated using the TRIzol reagent, as described by the manufacturer (15596018, Invitrogen). RNA-seq libraries were constructed and sequenced by Berry Genomics. RNA-seq NGS reads were aligned to the hg19 reference genome using the Mapsplice aligner (version 2.1.8) [75] with default parameters. The gene-level expression levels were quantified by RSEM (Version 1.2.19) [28,76], based on the TCGA mRNA-seq Pipeline (<https://webshare.bioinf.unc.edu/public/mRNAseq_TCGA/UNC_mRNAseq_summary.pdf>). Differentially expressed genes between samples were detected using EBSeq (Version 1.1.5) [77] and were defined as the PPDE over 0.99. Gene set enrichment analysis of KEGG pathways was performed using the Functional Annotation Tool from DAVID with default parameters [29,78]. Expression perturbations in significant KEGG pathways were determined by GAGE [30] with default parameters.

### **Statistical analyses**

Statistical analyses were performed using R. Student’s *t*-test and Wilcoxon test were used for calculating significance of between-group differences. Statistical significance is indicated by P < 0.05. All data were expressed as mean ± s.d. of at least three independent experiments.

## Data availability

The raw sequence data reported in this paper have been deposited in the Genome Sequence Archive in BIG Data Center, Beijing Institute of Genomics (BIG), Chinese Academy of Sciences, under accession numbers CRA001863, that are publicly accessible at http://bigd.big.ac.cn/gsa/s/d267AtvW.

## Code availability

Scripts for growth models are available at <https://github.com/TaoLee0510/>.

# References

60. Meissner JD. Nucleotide sequences and further characterization of human papillomavirus DNA present in the CaSki, SiHa and HeLa cervical carcinoma cell lines. *J Gen Virol* 1999;**80**:1725–33.

61. Hassell BYMP. Density-Dependence in Single-Species Populations. *J Anim Ecol* 1975;**44**:283–95.

62. Gompertz B, Transactions P, Society R. On the Nature of the Function Expressive of the Law of Human Mortality, and on a New Mode of Determining the Value of Life Contingencies. *Philos Trans ofthe R Soc London* 1825;**115**:513–8363. Verhulst PF. Recherches mathématiques sur la loi d’accroissement de la population. *Nouv mémoires l’Académie R des Sci B-lett Bruxelles* 1845:14–54.

64. Volterra V. Fluctuations in the abundance of a species considered mathematically. *Nature* 1926;**118**:558–60.

65. Bomze IM. Lotka-Volterra equation and replicator dynamics: A two-dimensional classification. *Biol Cybern* 1983;**48**:201–11.

66. Kaiser CA, Krieger M, Lodish H *et al.* *Molecular Cell Biology.* WH Freeman, 2007.

67. Boutros PC, Fraser M, Harding NJ *et al.* Spatial genomic heterogeneity within localized, multifocal prostate cancer. *Nat Genet* 2015;**47**:1–14.

68. Hao J-J, Lin D-C, Dinh HQ *et al.* Spatial intratumoral heterogeneity and temporal clonal evolution in esophageal squamous cell carcinoma. *Nat Genet* 2016;**48**:1500–7.

69. Gatenby RA, Silva AS, Gillies RJ *et al.* Adaptive therapy. *Cancer Res* 2009;**69**:4894–903.

70. Chen J, Sprouffske K, Huang Q *et al.* Solving the puzzle of metastasis: The evolution of cell migration in neoplasms. *PLoS One* 2011;**6**, DOI: 10.1371/journal.pone.0017933.

71. Tang L, Van De Ven AL, Guo D *et al.* Computational modeling of 3D tumor growth and angiogenesis for chemotherapy evaluation. *PLoS One* 2014;**9**:1–12.

72. Orlando PA, Gatenby RA, Brown JS. Tumor Evolution in Space: The Effects of Competition Colonization Tradeoffs on Tumor Invasion Dynamics. *Front Oncol* 2013;**3**:1–12.

73. Robertson-Tessi M, Gillies RJ, Gatenby RA *et al.* Impact of metabolic heterogeneity on tumor growth, invasion, and treatment outcomes. *Cancer Res* 2015;**75**:1567–79.

74. Degenhardt K, Mathew R, Beaudoin B *et al.* Autophagy promotes tumor cell survival and restricts necrosis, inflammation, and tumorigenesis. *Cancer Cell* 2006;**10**:51–64.

75. Wang K, Singh D, Zeng Z *et al.* MapSplice: Accurate mapping of RNA-seq reads for splice junction discovery. *Nucleic Acids Res* 2010;**38**:1–14.

76. Li B, Ruotti V, Stewart RM *et al.* RNA-Seq gene expression estimation with read mapping uncertainty. *Bioinformatics* 2009;**26**:493–500.

77. Leng N, Dawson JA, Thomson JA *et al.* EBSeq: An empirical Bayes hierarchical model for inference in RNA-seq experiments. *Bioinformatics* 2013;**29**:1035–43.

78. Huang DW, Sherman BT, Lempicki RA. Bioinformatics enrichment tools: Paths toward the comprehensive functional analysis of large gene lists. *Nucleic Acids Res* 2009;**37**:1–13.
