# Supplementary material for: Variation in the life history strategy underlies functional diversity of tumors
Source: Natl Sci Rev. 2020 Jun 5;8(2):nwaa124. doi: 10.1093/nsr/nwaa124 (PMC8288455; doi:10.1093/nsr/nwaa124)
Supplement: nwaa124_Supplement_File [file nwaa124_supplement_file.zip › Supplementary Figures and Tables revision_2nd.docx]

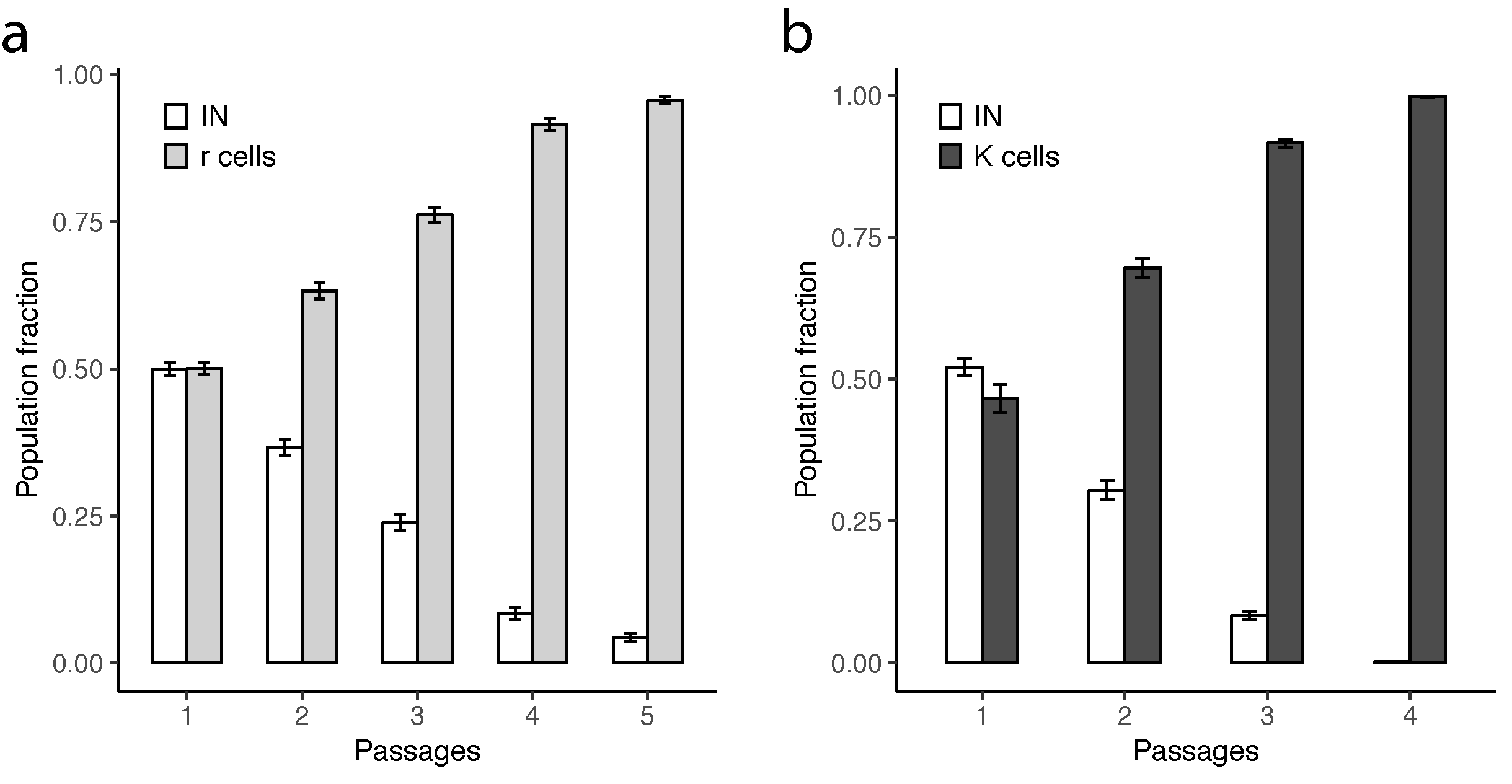


# Supplementary Figure 1 | Fitness of r and K cells compared with IN cells.

**IN cells and the a) r cells or b) K cells were mixed together at equal amounts at the beginning. The mixed populations were cultured under normal culture conditions.** The proportion of each type of cells was measured by flow cytometry every two days during subculture. Error bars represent standard deviations. N= 3 independent experiments per population, mean ± SD.


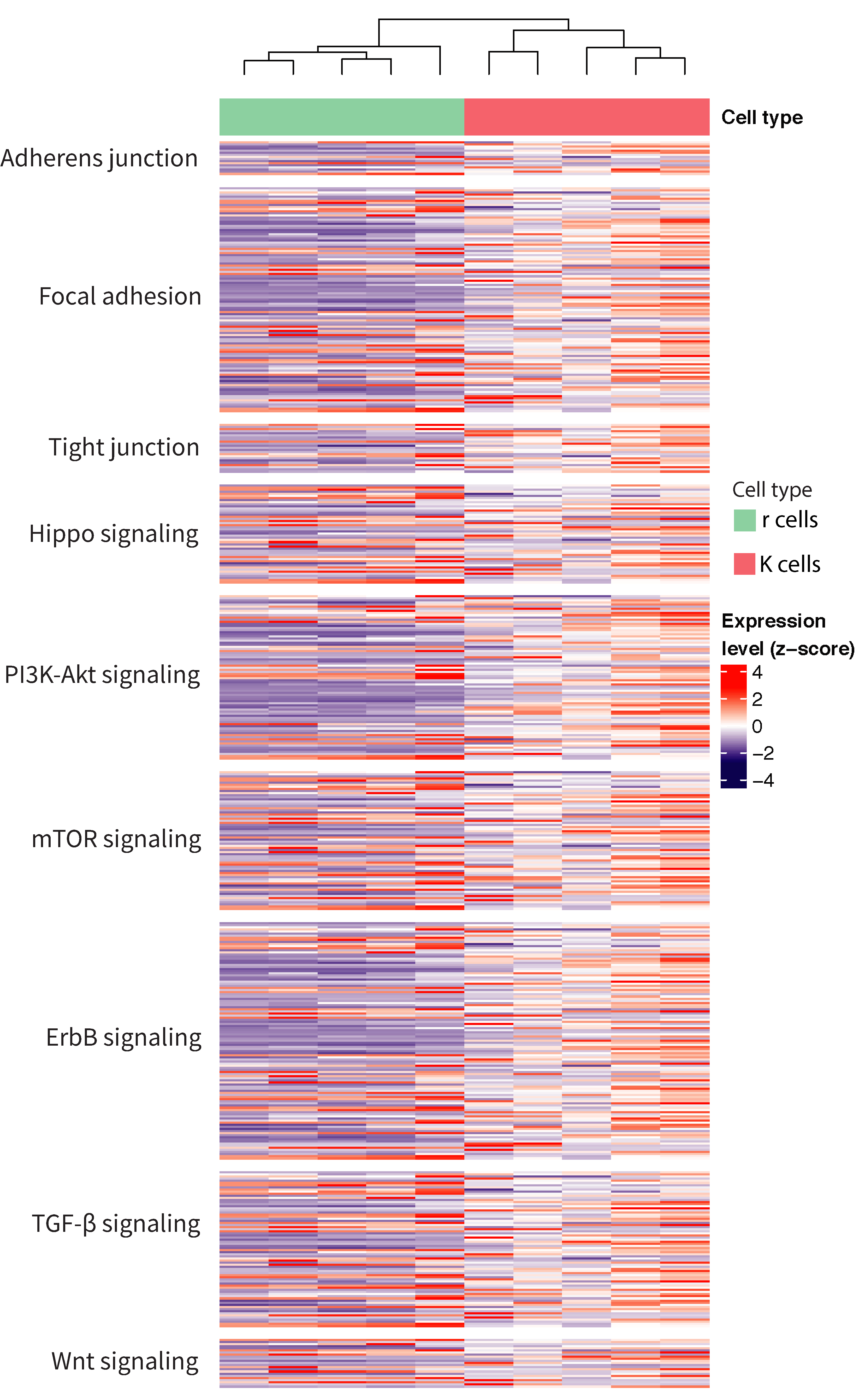


# Supplementary Figure 2 | Cluster of Hippo-related pathways.

The z-score heatmap indicates the scale of gene expression difference. The DEGs in 9 pathways which crosstalk with Hippo-Yap signaling pathway are used to the cluster analysis.

# Supplementary Figure 3 | Dlg-2 shRNA in K cells.

Dlg-2 shRNA in K cells. Three shRNAs were used for the Dlg-2 knockdown. The Y-axis represents the relative expression level. n=3 independent experiments per population, mean ± SD, Student's *t*-test: *P<0.05, **P<0.01.

**
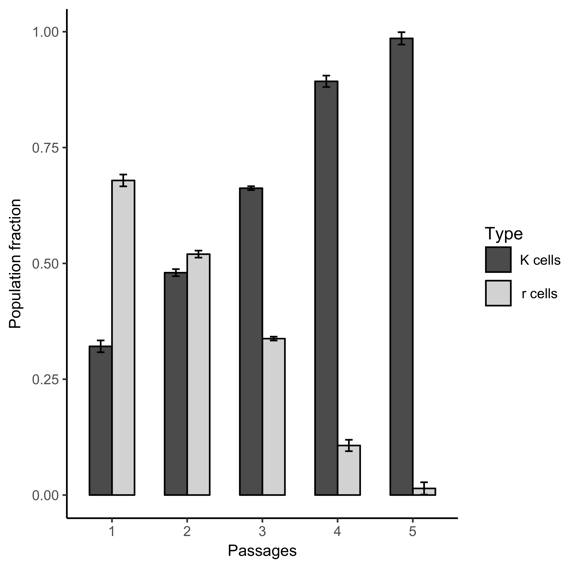
**

# Supplementary Figure 4 | Observed dynamics of mixed populations initiated with 90% r cells and 10% K cells.

Mixed populations were cultured under *K*-selection. The proportion of each type of cells was measured by flowcytometry every three days during. The bars represent the proportion change of cell population by time. The grey bars represent r and black bars K cells. The x-axis represents subculture times, the y-axis represents cell-type proportions. Three replicates were performed on each assay. Error bars represent standard deviations. n=3 independent experiments per population, mean ± SD.


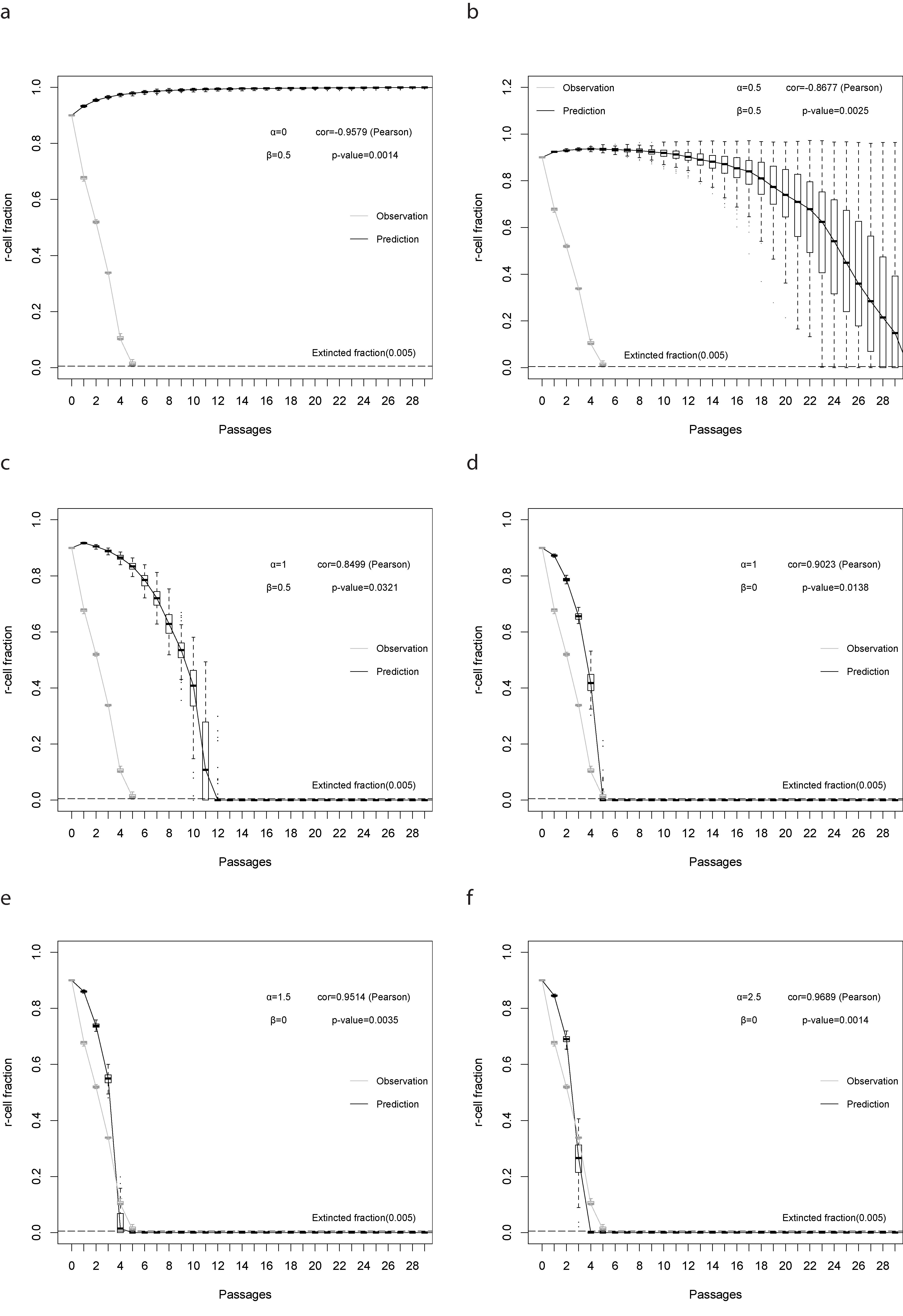


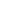

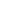


# Supplementary Figure 5 | Predicted dynamics of r and K cells mixed populations.

The proportion of each type of cells in the population was measured when subculturing. Sub-figures show the predicted population dynamics with different $\alpha$ and $\beta$. Black boxes and lines represent simulation results. Gray boxed and lines represent observations. n = 100 stochastic simulations per population, n= 3 independent experiments per population, mean ± SD.


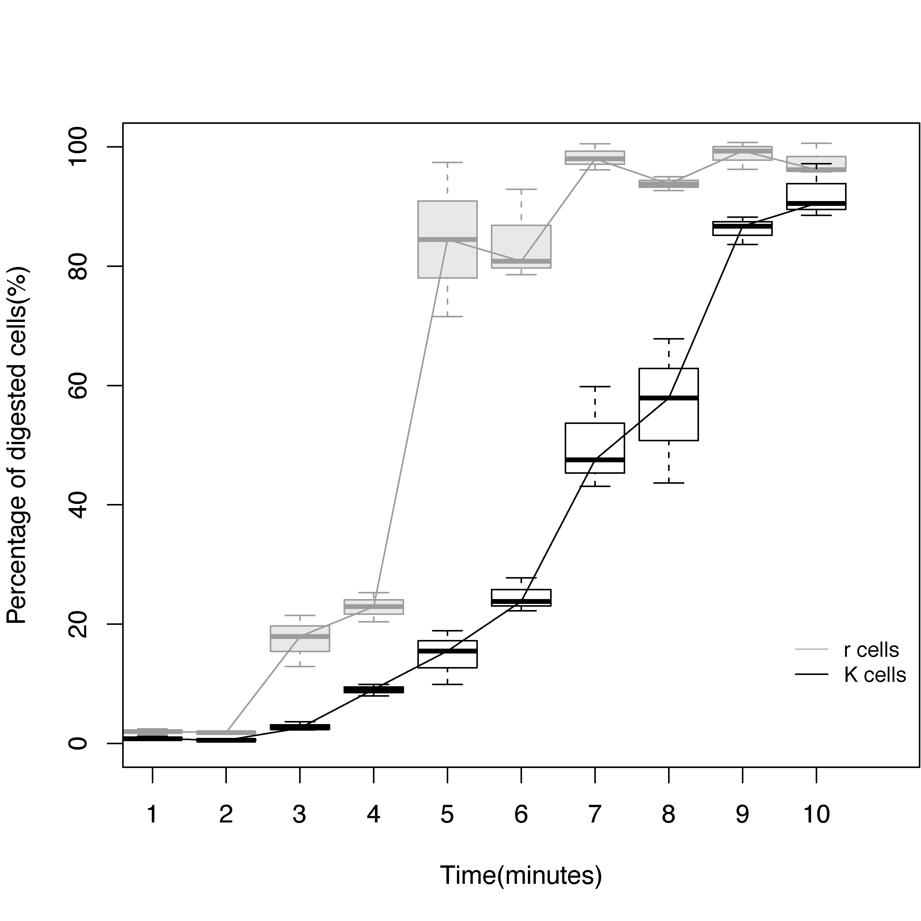


# Supplementary Figure 6 | Detachment curves of r and K cells under trypsinization.

Cells were digested by 1X Trypsin under room temperature. Cells which detached under trypsinization were counted every minute. The result shows that it takes significantly longer to digest attached K than r cells. The x-axis represents time and the y-axis represents the proportion of total cells that have been digested. Grey lines and box diagrams represent observations of r-cell populations. Black lines and box diagrams represent observations of K-cell populations. n= 6 independent experiments per population, mean ± SD.


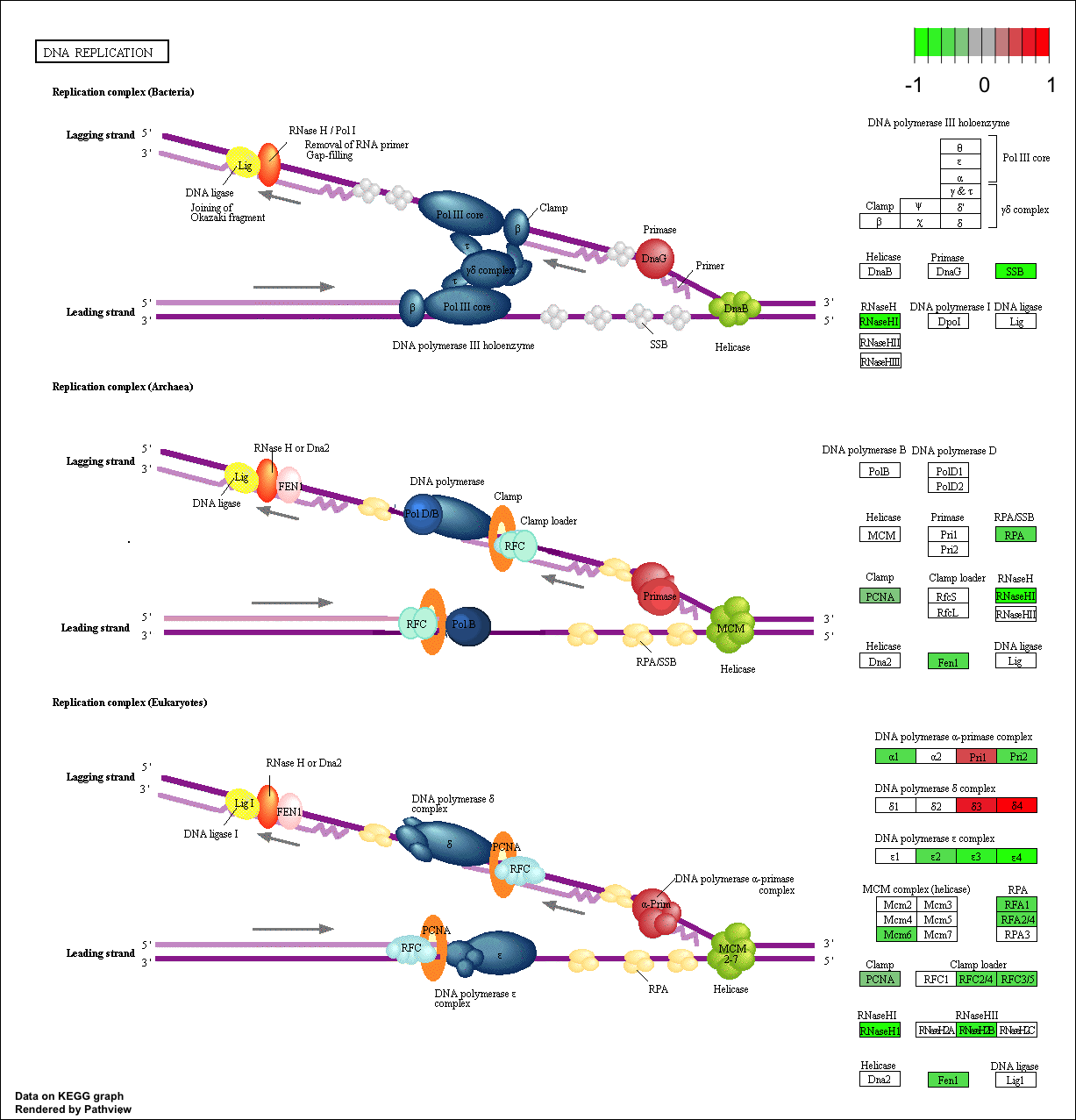


# Supplementary Figure 7 | The DNA replication pathway is significantly lower expressed in r cells under high than under low density.

The high expressed genes are marked with red and the low expressed genes are marked with green.


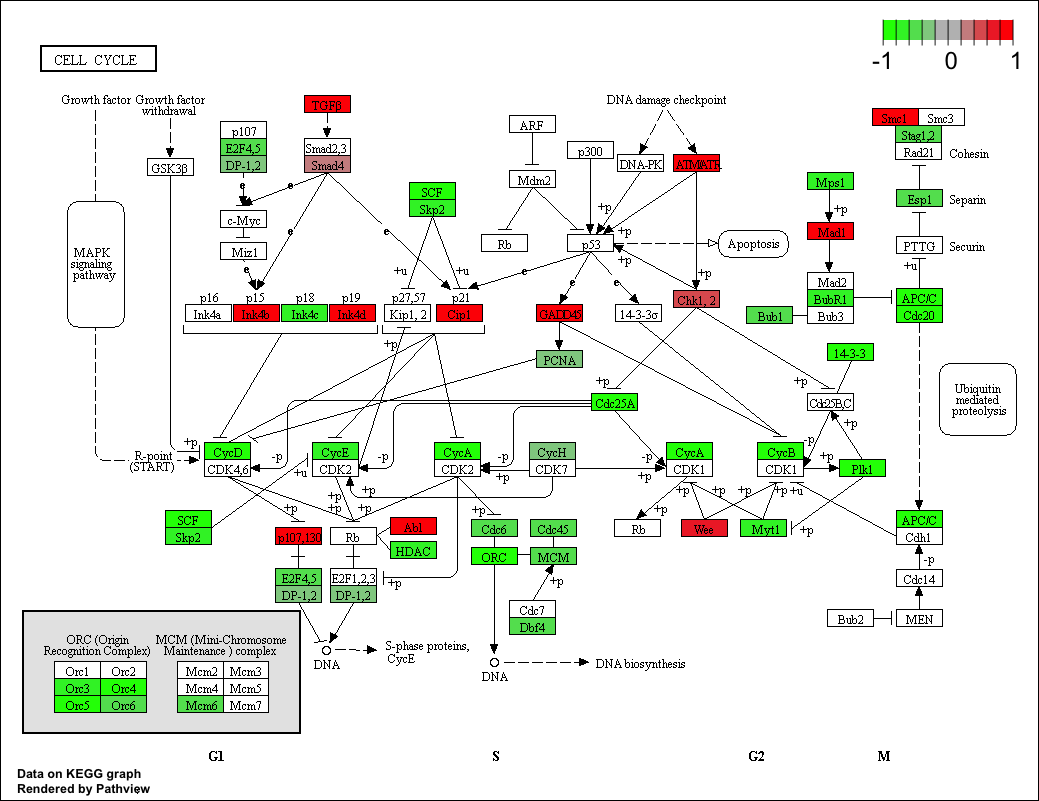


# Supplementary Figure 8 | The cell cycle pathway is significantly lower expressed in r cells under high than under low density.

The high expressed genes are marked with red and the low expressed genes are marked with green.


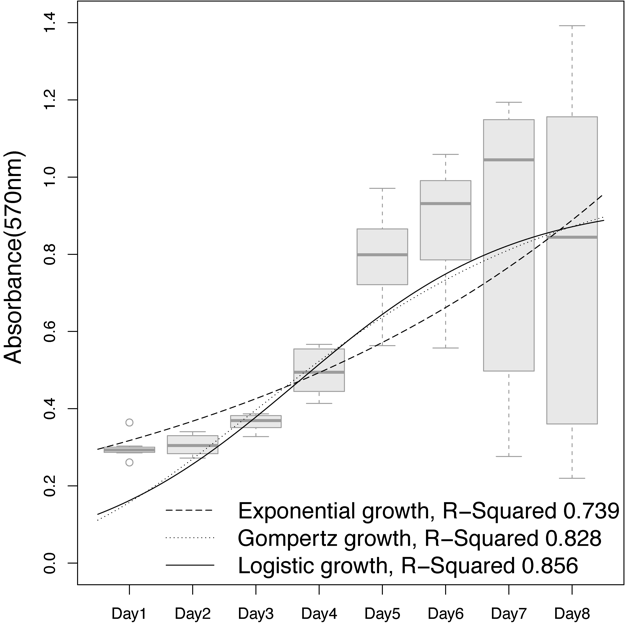


# Supplementary Figure 9 | Growth model fitting.

Cell growth was calculated using the MTT cell proliferation assay. We take the absorbance at 570 nm as the relative cell number. The assay was performed over eight days. We chose three population growth models: Exponential (R- Squared number 0.739; p=3.02e-05), Gompertz (R- Squared number 0.828; p=7.7e-05)) and Logistic (R- Squared number 0.856; p=4.95e-05). Curves represent different models’ predictions.

# Supplementary Figure 10 | Carrying capacity estimation.

The X-axis represents days after cell seeding. The Y-axis represents cell density. The units of ell density are number of cells per square centimeter. Grey points represent the cell density of r and black points of K cells. Data were collected from experiments. Solid grey and solid black lines represent estimated growth curves of r and K cell populations respectively. The functions of density curve of r and K cells were estimated as 228280/(1+83.485 exp(-0.80585 x)) and 239120/(1+728.8 exp(-1.0549 x)), respectively.


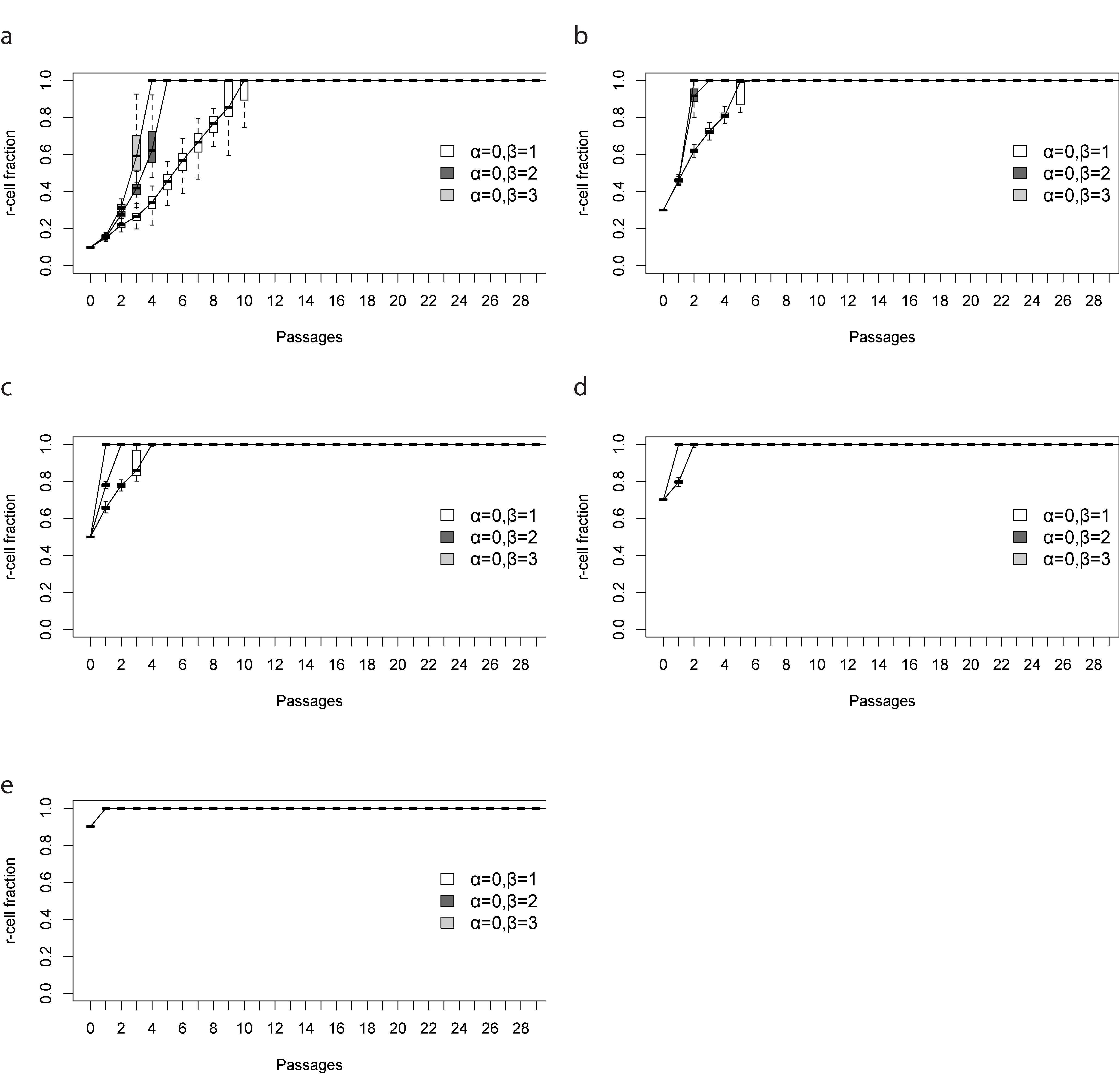


# Supplementary Figure 11 | Predicted dynamics of r and K cells mixed populations.

**The mixed populations were cultured under high density based on the density dependent population growth model.** The populations were initialed with cell density of $4\times{10}^{4} cells/\mathrm{cm}^{2}$ and subcultured every 72 hours. **The proportion of r cells is a) 10%, b) 30%, c) 50%, d) 70% e) 90% at the beginning.** The proportion of each type of cells in a population was measured when subculturing. n = 100 stochastic simulations per population; mean ± SD.


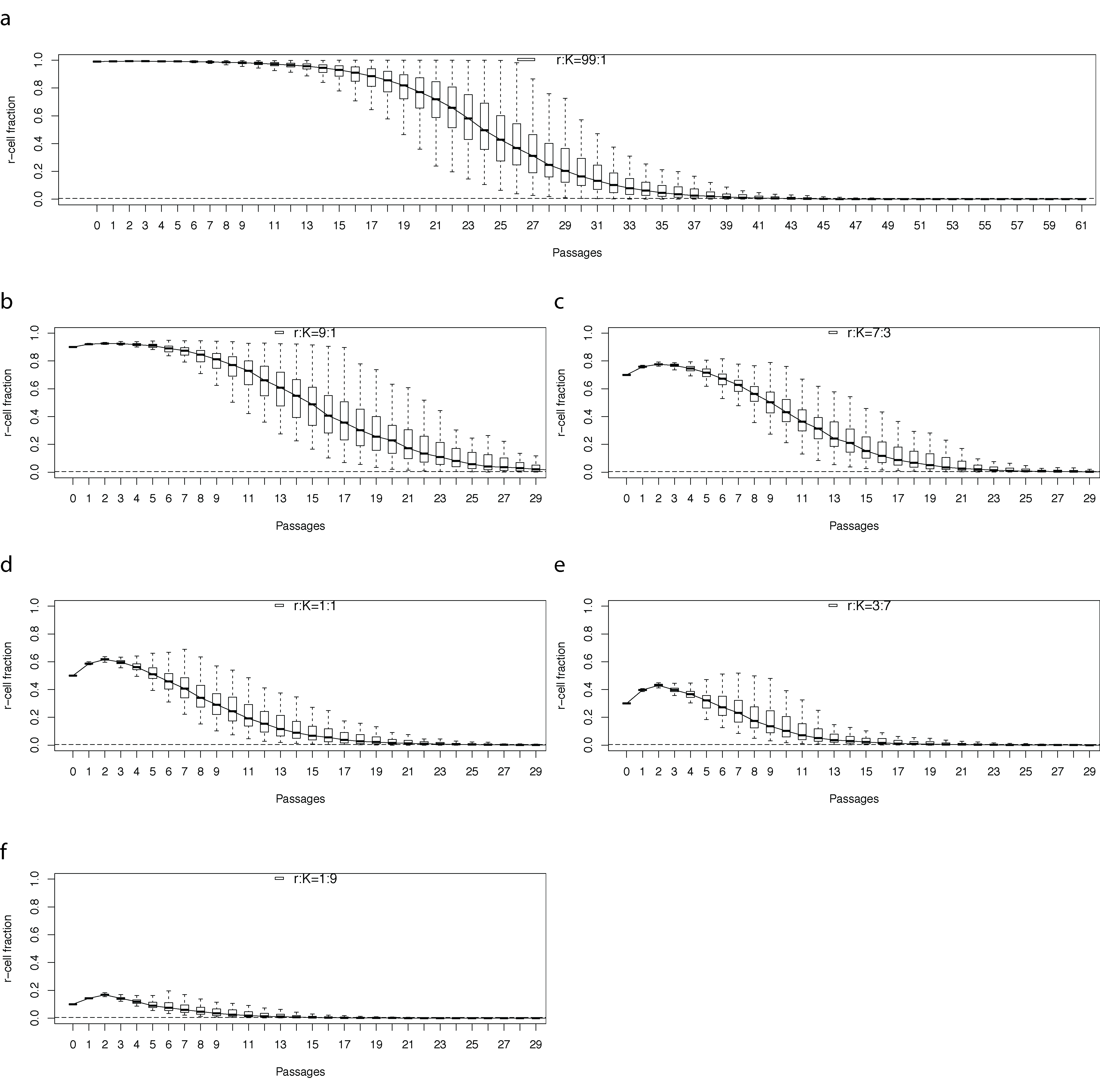


# Supplementary Figure 12 | The dynamics of r and K cell mixture populations.

Each panel shows 100 simulation predictions of a mixture population with a certain initial r and K cells ratio. The x-axis represents the passage times and the y-axis represents the r-cell fraction. n = 100 stochastic simulations per population; mean ± SD.


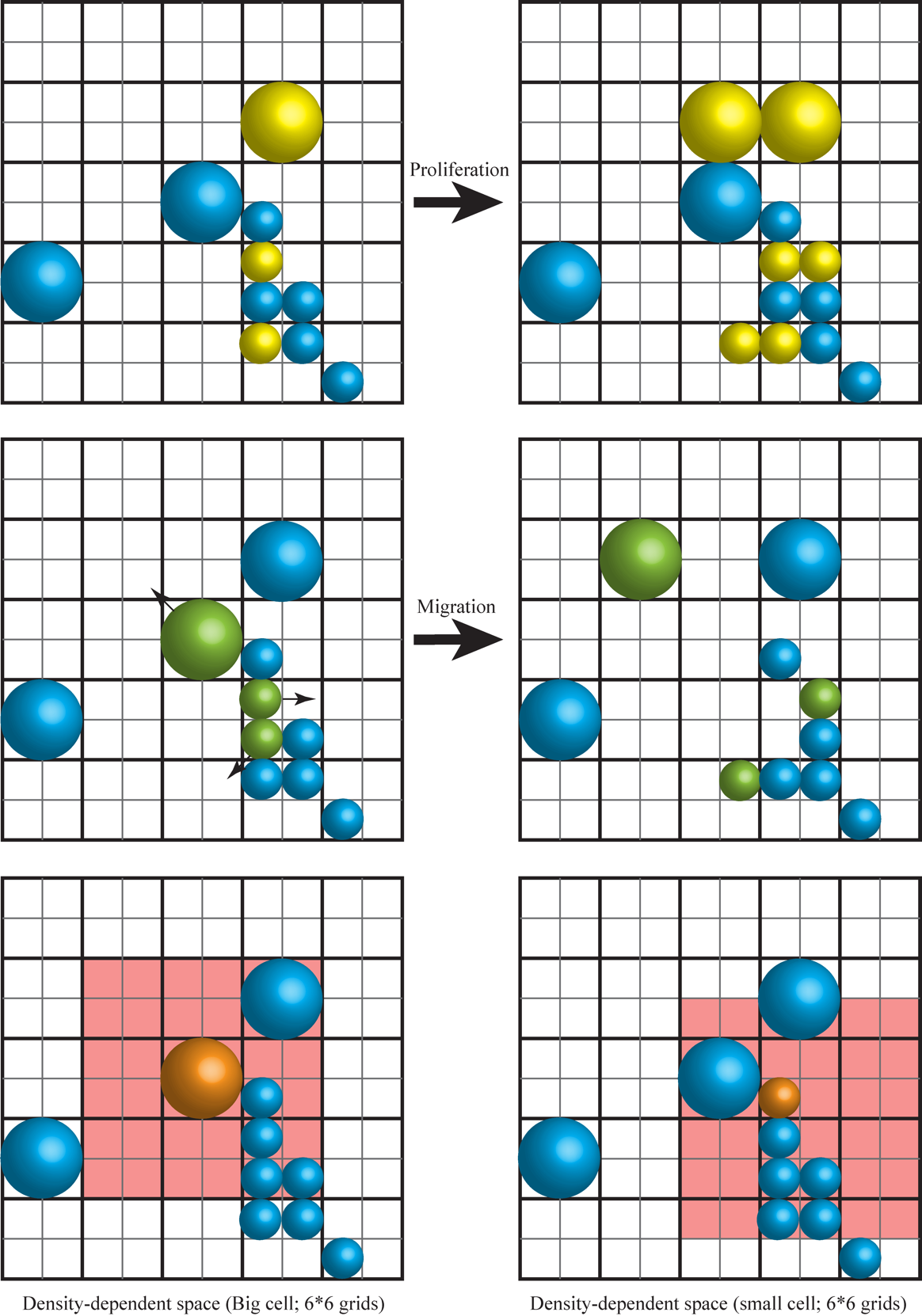


# Supplementary Figure 13 | The spatial computational model of population growth.

The cell growth space was assumed to be a two-dimensional planar grid. The location of cells is determined by grid coordinates. Cell migration and division are on the two-dimensional grid plane. The first line represents the division process. Yellow cells are undergoing mitosis. The second row represents migration with migrated cells in green. The red regions in the third row represent density dependent regions of migrated cells (orange).


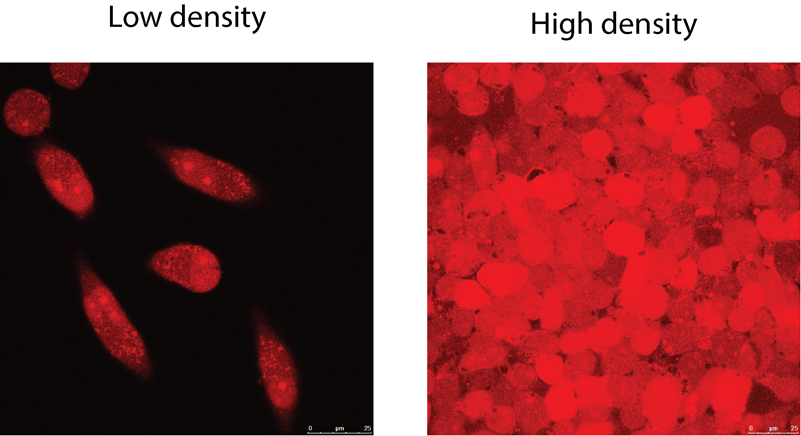


# Supplementary Figure 14 | Density-dependent cell size.

Fluorescence imaging of cells at two densities. Red marks cell bodies. On the left is the image of cells growing under low density and on the right under high.


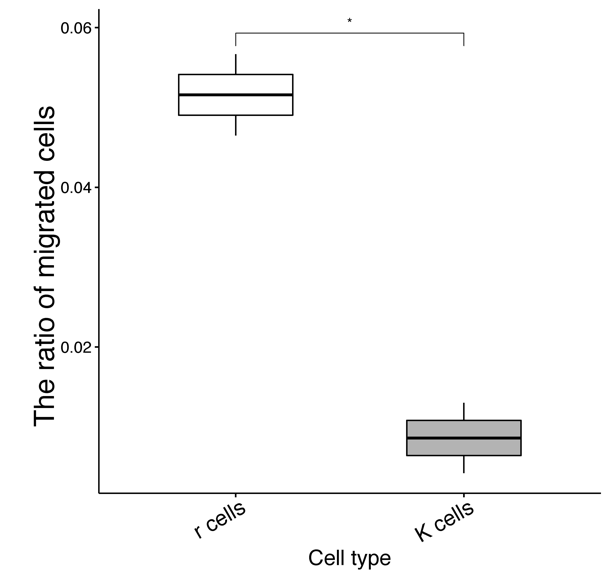


# Supplementary Figure 15 | Ratio of migrated cells.

r cells migrate more readily than K cells (t-test). The data were collected using a trans-well migration assay. n = 6 independent experiments; mean ± SD, Student's *t*-test: *P<0.05.

# Supplementary Figure 16 | α and β estimation.

The heatmap showed the Pearson correlation coefficient and the p-value between the observations and the predictions with different value of α and β. *:p<0.05, **:p<0.01,***:p<0.001.

# Supplementary Table 1 | The number of DEGs across comparisons.

| **Comparisons** | **High-expressed genes number** | **Low-expressed genes number** | **Total DEGs number** |
| --- | --- | --- | --- |
| KL vs. rL | 1748 | 1413 | 3161 |
| KH vs. KL | 1151 | 1126 | 2277 |
| rH vs. rL | 3284 | 3098 | 6382 |

Note: KL: K cells under low density condition; KH: K cells under high density condition; rL: r cells under low density condition; rH: r cells under high density condition.

# Supplementary Table 2 | Enrichment of DEGs in r and K cells under low-density.

| **KEGG Pathway** | **Count** | **%** | **P-Value** |
| --- | --- | --- | --- |
| Spliceosome | 54 | 1.7 | 1.00E-10 |
| Pathways in cancer | 97 | 3.1 | 2.90E-05 |
| Ribosome biogenesis in eukaryotes | 30 | 1 | 9.70E-05 |
| Small cell lung cancer | 29 | 0.9 | 1.60E-04 |
| Hepatitis B | 40 | 1.3 | 1.10E-03 |
| PI3K-Akt signaling pathway | 79 | 2.5 | 2.00E-03 |
| ECM-receptor interaction | 26 | 0.8 | 3.20E-03 |
| RNA transport | 43 | 1.4 | 5.20E-03 |
| Proteasome | 15 | 0.5 | 9.50E-03 |
| Base excision repair | 12 | 0.4 | 1.40E-02 |
| p53 signaling pathway | 19 | 0.6 | 2.30E-02 |
| Amoebiasis | 27 | 0.9 | 2.30E-02 |
| Epstein-Barr virus infection | 43 | 1.4 | 2.80E-02 |
| Pyrimidine metabolism | 26 | 0.8 | 3.20E-02 |
| Hippo signaling pathway | 35 | 1.1 | 3.50E-02 |
| Axon guidance | 30 | 1 | 4.20E-02 |
| Cell cycle | 29 | 0.9 | 5.10E-02 |
| Arginine and proline metabolism | 14 | 0.4 | 6.10E-02 |
| Amyotrophic lateral sclerosis (ALS) | 14 | 0.4 | 6.10E-02 |
| Influenza A | 38 | 1.2 | 6.30E-02 |
| Pertussis | 19 | 0.6 | 6.30E-02 |
| AMPK signaling pathway | 28 | 0.9 | 6.70E-02 |
| Purine metabolism | 38 | 1.2 | 7.20E-02 |
| RNA degradation | 19 | 0.6 | 7.80E-02 |
| Complement and coagulation cascades | 17 | 0.5 | 9.90E-02 |

# Supplementary Table 3 | Top 25 pathways enriched in r and K cells under crowed culture.

| **Term** | **Count** | **%** | **PValue** | **Fold Enrichment** |
| --- | --- | --- | --- | --- |
| Proteasome | 19 | 1.07344633 | 9.32E-09 | 4.89157973 |
| Spliceosome | 34 | 1.92090395 | 2.58E-08 | 2.89584617 |
| RNA transport | 34 | 1.92090395 | 1.30E-05 | 2.23922989 |
| Oxidative phosphorylation | 26 | 1.46892655 | 2.03E-04 | 2.2144706 |
| Alzheimer's disease | 30 | 1.69491525 | 3.02E-04 | 2.02283372 |
| Ribosome biogenesis in eukaryotes | 19 | 1.07344633 | 4.70E-04 | 2.47390239 |
| Huntington's disease | 32 | 1.8079096 | 6.32E-04 | 1.88797814 |
| Parkinson's disease | 24 | 1.3559322 | 0.00293437 | 1.91456938 |
| Non-alcoholic fatty liver disease (NAFLD) | 25 | 1.41242938 | 0.00310229 | 1.87547498 |
| Epstein-Barr virus infection | 29 | 1.63841808 | 0.00448698 | 1.72899051 |
| RNA polymerase | 9 | 0.50847458 | 0.00543855 | 3.18596311 |
| mRNA surveillance pathway | 17 | 0.96045198 | 0.00545382 | 2.11619528 |
| RNA degradation | 15 | 0.84745763 | 0.00669615 | 2.2067277 |
| Ribosome | 22 | 1.24293785 | 0.00765604 | 1.83244937 |
| Pyrimidine metabolism | 18 | 1.01694915 | 0.00895992 | 1.96059269 |
| Lysosome | 19 | 1.07344633 | 0.01859407 | 1.77875627 |
| Cytosolic DNA-sensing pathway | 12 | 0.6779661 | 0.02280654 | 2.12397541 |
| Metabolic pathways | 128 | 7.23163842 | 0.02350298 | 1.18075506 |
| Herpes simplex infection | 25 | 1.41242938 | 0.03073077 | 1.54752307 |
| p53 signaling pathway | 12 | 0.6779661 | 0.03105377 | 2.02887203 |
| Protein export | 6 | 0.33898305 | 0.04640999 | 2.95509622 |
| Purine metabolism | 23 | 1.29943503 | 0.05854707 | 1.4803465 |
| Protein processing in endoplasmic reticulum | 22 | 1.24293785 | 0.0668094 | 1.47463382 |
| Lysine degradation | 9 | 0.50847458 | 0.08312794 | 1.96059269 |
| Synaptic vesicle cycle | 10 | 0.56497175 | 0.09925729 | 1.79807442 |

# Supplementary Table 4 | The enrichment of the common DEGs in r and K cells under high-density stress.$\alpha and \beta estimation.$

| **Gene expression change** | **Go Term and KEGG Pathway** | **P-value** |
| --- | --- | --- |
| Up regulated | GO:0001666~response to hypoxia | 0.002421258 |
|  | GO:0042981~regulation of apoptotic process | 0.011527814 |
|  | GO:0043065~positive regulation of apoptotic process | 0.011982769 |
|  | GO:0071456~cellular response to hypoxia | 0.014334497 |
|  | hsa04512: ECM-receptor interaction | 1.86E-04 |
|  | hsa04510: Focal adhesion | 0.016490211 |
|  | hsa01200: Carbon metabolism | 0.039001084 |
| Down regulated | GO:0043488~regulation of mRNA stability | 4.38E-24 |
|  | GO:0006521~regulation of cellular amino acid metabolic process | 1.30E-20 |
|  | GO:0051436~negative regulation of ubiquitin-protein ligase activity involved in mitotic cell cycle | 2.36E-19 |
|  | GO:0032981~mitochondrial respiratory chain complex I assembly | 3.99E-14 |
|  | GO:0051301~cell division | 1.47E-06 |
|  | hsa04110: Cell cycle | 5.64E-04 |
|  | hsa00020: Citrate cycle (TCA cycle) | 9.05E-04 |
|  | hsa01100: Metabolic pathways | 0.001216333 |


# Supplementary Table 5 |$\mathbf{Single cell growth rate.}$

| **Samples** | **IN_G** | **IN_R** | **G3K** | **R1K** | **G3r** | **R1r** |
| --- | --- | --- | --- | --- | --- | --- |
| 1 | 1.18205451 | 0.9099664 | 1.05785739 | 0.4169925 | 1.1529676 | 1.36096405 |
| 2 | 0.78450625 | 1.09276245 | 0.96578718 | 0.3 | 0.63894989 | 1.20447356 |
| 3 | 0.68331135 | 0.92571359 | 0.40356018 | 1.20420227 | 1.35024631 | 1.26211361 |
| 4 | 0.96146344 | 0.37275673 | 0.96610808 | 0.75038257 | 1.26650438 | 1.29541963 |
| 5 | 0.69223035 | 0.97243412 | 1.17496324 | 0.73516754 | 1.24553979 | 1.35627194 |
| 6 | 0.55066724 | 0.96347845 | 0.10820526 | 1.06598844 | 1.29776714 | 1.27910611 |
| 7 | 1.11262124 | 0.65023927 | 0.64227275 | 1.33353816 | 1.3162349 | 1.29989319 |
| 8 | 0.78234633 | 0.32142857 | 0.62534911 | 0.0830075 | 1.34890222 | 1.22927817 |
| 9 | 0.85056211 | 0.60949718 | 0.40645792 | 1.2428491 | 1.06745604 | 1.25850872 |
| 10 | 0.97209214 | 0.78990066 | 0.89551763 | 1 | 1.19063932 | 1.31476819 |
| 11 | 0.69328098 | 0.09441166 | 0.3050203 | 0.29657843 | 1.29025241 | 0.74968538 |
| 12 | 0.28188874 | 0.9669731 | 1.22090362 | 0.1169925 | 1.35733656 | 1.27015772 |
| 13 | 1.06924641 | 0.93169468 | 0.03571429 | 0.14150375 | 1.27256068 | 1.34576374 |
| 14 | 0.83532878 | 0.86647011 | 0.59787896 | 0.53219281 | 1.00358289 | 1.32127623 |
| 15 | 0.90082758 | 0.95006167 | 0.90651607 | 1.15065555 | 1.24181886 | 1.3181352 |
| 16 | 1.12741936 | 1.16930541 | 0.38410313 | 1.09248125 | 1.34462084 | 1.38411497 |
| 17 | 0.94026379 | 1.01658765 | 0.16374866 | 1.03257973 | 1.11478234 | 1.09955316 |
| 18 | 1.22249553 | 1.03685187 | 1.13752093 | 0.99068906 | 1.24553253 | 1.51453513 |
| 19 | 0.70769319 | 0.99006821 | 0.52363493 | 1.11065629 | 1.25292585 | 1.14394413 |
| 20 | 0.3090178 | 0.57221968 | 0.69534322 | 0.95216004 | 1.20452535 | 0.80808175 |
| 21 | 0.20838377 | 1.06415693 | 0.74422446 | 1.25041672 | 1.3004986 | 0.35686687 |
| 22 | 1.04414208 | 0.71627403 | 0.10714286 | 1 | 1.20976265 | 1.34431378 |
| 23 | 0.81863137 | 1.01558597 | 0.3187433 | 1.18371023 | 0.5992702 | 0.9137845 |
| 24 | 0.77428678 | 0.69809307 | 0.98152095 | 0.79366379 | 1.19494849 | 1.38846475 |
| 25 | 0.95431981 | 0.61747098 | 1.14700834 | 0.37598882 | 1.11092661 | 1.49837062 |
| 26 | 1.06200509 | 1.05243698 | 1.04377307 | 0.1169925 | 0.11321161 | 1.45951409 |
| 27 | 1.17748589 | 0.85243595 | 1.04125522 | 0.35108335 | 1.21091295 | 1.26717622 |
| 28 | 1.02449193 | 0.9846867 | 0.35633143 | 0.54431378 | 1.23501737 | 1.43353816 |
| 29 | 0.70446769 | 0.82035446 | 0.56899428 | 0.19967234 | 1.23319832 | 0.45395466 |
| 30 | 0.65930187 | 0.68832903 | 1.04866898 | 0.99772799 | 1.32590857 | 0.2 |
| 31 | 0.8158403 | 0.55060698 | 0.33996806 | 0.15849625 | 0.8263962 | 0.62407913 |
| 32 | 0.92821544 | 1.03767951 | 1.20698559 | 0.5 | 0.11595502 | 1.50891297 |
| 33 | 0.90263343 | 0.95049219 | 0.89434196 | 0.2299685 | 1.23633227 | 0.62419087 |
| 34 | 0.74990959 | 0.40670889 | 0.46312742 | 0.9194603 | 1.07652077 | 1.37139771 |
| 35 | 1.30573712 | 0.86764158 | 0.98416201 | 0.1169925 | 1.29667389 | 1.21301415 |
| 36 | 0.57407648 | 1.00402174 | 0.77471437 | 1.19858419 | 0.97046398 | 1.39163564 |
| 37 | 0.69373464 | 1.08973541 | 1.26907276 | 1.06438562 | 1.30892878 | 1.42041182 |
| 38 | 0.86274954 | 0.57086811 | 0.37654 | 1.23151496 | 1.30859543 | 1.28812369 |
| 39 | 1.11052423 | 0.19070893 | 0.80118862 | 1.1019417 | 1.11123277 | 1.30429845 |
| 40 | 1.16363931 | 0.68239273 | 0.73597283 | 0.59188632 | 1.34714326 | 1.24576374 |
| 41 | 0.31025775 | 0.70440812 | 1.17714937 | 1.09858419 | 1.30414223 | 1.06800298 |
| 42 | 1.09899887 | 1.03028933 | 0.93832419 | 0.9509775 | 0.83086675 | 1.30887882 |
| 43 | 1.02120064 | 0.77007585 | 0.9203289 | 0.40808175 | 1.33986971 | 1.53174597 |
| 44 | 1.06970496 | 0.75111087 | 0.87874437 | 0.2 | 0.31939515 | 1.49887069 |
| 45 | 0.41965783 | 0.75696406 | 0.7838964 | 1.12753486 | 1.20365572 | 0.38846475 |
| 46 | 0.68318868 | 1.00157957 | 0.94014636 | 0.5169925 | 1.30990997 | 1.42316337 |
| 47 | 1.11510448 | 0.97776 | 0.51172694 | 0.75038257 | 1.18687715 | 1.21357093 |
| 48 | 0.35658708 | 0.78473438 | 0.9950518 | 0.3 | 1.20095467 | 0.44576374 |
| 49 | 0.5841186 | 0.96386567 | 0.32020658 | 0.26767629 | 1.34738565 | 1.2258566 |
| 50 | 0.6865186 | 0.26844754 | 0.21428571 | 0.933985 | 0.94737447 | 0.67234 |
| 51 | 0.99740186 | 0.23728058 | 0.02696027 | 0.8 | 1.14735149 | 0.9950518 |
| 52 | 0.2343992 | 1.26351184 | 1.04299544 | 0.8 | 0.78302047 | 1.15481502 |
| 53 | 0.16042332 | 0.78684039 | 1.11587849 | 0.99083926 | 1.12030475 | 0.34330054 |
| 54 | 0.81002746 | 0.60953941 | 0.21428571 | 0.81032878 | 0.2947848 | ---- |
| 55 | 0.28893487 | 1.07336619 | 0.14285714 | 0.90947375 | ---- | ---- |
| 56 | ---- | 0.99543854 | 0.95528537 | 0.96232719 | ---- | ---- |
| 57 | ---- | 0.81517588 | 0.10714286 | 0.3 | ---- | ---- |
| 58 | ---- | 1.26762588 | 0.92306393 | 1.06219946 | ---- | ---- |
| 59 | ---- | 0.60449721 | 0.94750032 | 0.88137812 | ---- | ---- |
| 60 | ---- | 0.9382225 | 0.19946295 | 1.11357093 | ---- | ---- |
| 61 | ---- | 0.68801814 | 0.19946295 | 0.96211361 | ---- | ---- |
| 62 | ---- | 1.00723175 | 0.21428571 | 0.04150375 | ---- | ---- |
| 63 | ---- | 0.73484628 | 0.33963482 | 1.26800298 | ---- | ---- |
| 64 | ---- | 0.62672214 | 0.67506152 | 0.51083347 | ---- | ---- |
| 65 | ---- | 0.82513271 | 0.36921685 | 0.05849625 | ---- | ---- |
| 66 | ---- | 0.85640135 | ---- | 0.0509775 | ---- | ---- |
| 67 | ---- | 0.94903135 | ---- | 0.2 | ---- | ---- |
| 68 | ---- | 0.78134801 | ---- | 0.12288187 | ---- | ---- |
| 69 | ---- | 0.09838884 | ---- | 0.82288187 | ---- | ---- |
| 70 | ---- | 0.80426275 | ---- | 0.76617781 | ---- | ---- |
| 71 | ---- | 0.26280965 | ---- | 0.3 | ---- | ---- |
| 72 | ---- | ---- | ---- | 0.69858419 | ---- | ---- |
| 73 | ---- | ---- | ---- | 0.56438562 | ---- | ---- |
| 74 | ---- | ---- | ---- | 0.51083347 | ---- | ---- |
| 75 | ---- | ---- | ---- | 0.69645165 | ---- | ---- |
| 76 | ---- | ---- | ---- | 0.39138389 | ---- | ---- |

Note:IN_G: Initial cells with eGFP; IN_R: Initial cells with dsRed; G3K/G3r: K cells/ r cells with eGFP(the 3^rd^ replicates); R3K/R3r: K cells/ r cells with dsRed(the 3^rd^ replicates).

# Supplementary Table 6 | The abbravations of KEGG patways in Figure 2d

| **Abbravation** | **Pathway** |
| --- | --- |
| AJ | Adherens junction |
| AXG | Axon guidance |
| BER | Base excision repair |
| CAM | Cell adhesion molecules (CAMs) |
| CC | Cell cycle |
| CCC | Complement and coagulation cascades |
| CDP | Cytosolic DNA-sensing pathway |
| ECM | ECM-receptor interaction |
| ERBB | ErbB signaling pathway |
| FA | Focal adhesion |
| GM | Glycerophospholipid metabolism |
| HEL | Hematopoietic cell lineage |
| ISL | Insulin signaling pathway |
| JST | Jak-STAT signaling pathway |
| LD | Lysine degradation |
| LTM | Leukocyte transendothelial migration |
| LTP | Long-term potentiation |
| MAPK | MAPK signaling pathway |
| NCMC | Natural killer cell mediated cytotoxicity |
| NSP | Neurotrophin signaling pathway |
| OD | Osteoclast differentiation |
| OM | Oocyte meiosis |
| PDA | Protein digestion and absorption |
| PGS | Phagosome |
| POM | Progesterone-mediated oocyte maturation |
| POS | Peroxisome |
| PPAR | PPAR signaling pathway |
| PPER | Protein processing in endoplasmic reticulum |
| PTS | Proteasome |
| PUM | Purine metabolism |
| PYM | Pyrimidine metabolism |
| RAC | Regulation of actin cytoskeleton |
| RBE | Ribosome biogenesis in eukaryotes |
| RD | RNA degradation |
| RSP | mRNA surveillance pathway |
| RT | RNA transport |
| SPS | Spliceosome |
| TGFB | TGF-beta signaling pathway |
| TLR | Toll-like receptor signaling pathway |
| UMP | Ubiquitin mediated proteolysis |
